# Murine gut microbial interactions exert antihyperglycemic effects

**DOI:** 10.1093/ismejo/wraf028

**Published:** 2025-02-17

**Authors:** Liying Guo, Libing Xu, Yanhong Nie, Lu Liu, Zongping Liu, Yunpeng Yang

**Affiliations:** Jiangsu Co-innovation Center for Prevention and Control of Important Animal Infectious Diseases and Zoonoses, College of Veterinary Medicine, Yangzhou University, Yangzhou 225009, China; Institute of Comparative Medicine, Yangzhou University, Yangzhou 225009, China; Jiangsu Co-innovation Center for Prevention and Control of Important Animal Infectious Diseases and Zoonoses, College of Veterinary Medicine, Yangzhou University, Yangzhou 225009, China; Institute of Comparative Medicine, Yangzhou University, Yangzhou 225009, China; Shanghai Center for Brain Science and Brain-Inspired Technology, Shanghai 201602, China; Institute of Neuroscience, CAS Key Laboratory of Primate Neurobiology, State Key Laboratory of Neuroscience, CAS Center for Excellence in Brain Science and Intelligence Technology, Chinese Academy of Sciences, Shanghai 200031, China; Key Laboratory of Genetic Evolution & Animal Models, Kunming Institute of Zoology, Chinese Academy of Sciences, Kunming, Yunnan 650223, China; Shanghai Center for Brain Science and Brain-Inspired Technology, Shanghai 201602, China; Institute of Neuroscience, CAS Key Laboratory of Primate Neurobiology, State Key Laboratory of Neuroscience, CAS Center for Excellence in Brain Science and Intelligence Technology, Chinese Academy of Sciences, Shanghai 200031, China; Key Laboratory of Genetic Evolution & Animal Models, Kunming Institute of Zoology, Chinese Academy of Sciences, Kunming, Yunnan 650223, China; Jiangsu Co-innovation Center for Prevention and Control of Important Animal Infectious Diseases and Zoonoses, College of Veterinary Medicine, Yangzhou University, Yangzhou 225009, China; Institute of Comparative Medicine, Yangzhou University, Yangzhou 225009, China; Jiangsu Co-innovation Center for Prevention and Control of Important Animal Infectious Diseases and Zoonoses, College of Veterinary Medicine, Yangzhou University, Yangzhou 225009, China; Institute of Comparative Medicine, Yangzhou University, Yangzhou 225009, China; Shanghai Center for Brain Science and Brain-Inspired Technology, Shanghai 201602, China; Institute of Neuroscience, CAS Key Laboratory of Primate Neurobiology, State Key Laboratory of Neuroscience, CAS Center for Excellence in Brain Science and Intelligence Technology, Chinese Academy of Sciences, Shanghai 200031, China

**Keywords:** *Escherichia coli* Nissle 1917, bacteriocin, gut microbial interactions, glucose metabolism, hyperglycemia

## Abstract

The correlations between gut microbiota and host metabolism have been studied extensively, whereas little relevant work has been done to investigate the impact of gut microbial interactions on host metabolism. With the use of a bacteriocin-targeting strategy, we aimed to identify the gut microbes associated with glucose and lipid metabolism by adjusting the gut microbial composition of mice fed a high-fat diet. To fulfill this goal, a *Listeria monocytogenes* (Lmo)-derived bacteriocin Lmo2776 secretion module was constructed and integrated into the genome of *Escherichia coli* Nissle 1917 (EcN), yielding the Lmo2776-secreting strain EcN-2776. In high-fat diet-fed mice, EcN-2776 administration decreased blood glucose and increased serum triglyceride, and gene amplicon sequencing of 16S rRNA in these mice indicated that intestinal secretion of Lmo2776 led to adjustment of the gut microbial composition. Specifically, Lmo2776 restricted the growth of *Ligilactobacillus murinus*, thus alleviating its inhibitory impact towards *Faecalibaculum rodentium*. Further analyses indicated that *F. rodentium* administration decreased the fasting blood glucose of high-fat diet-fed mice, an effect that may be attributable to the intestinal consumption of glucose by *F. rodentium*. In this study, we identified the gut microbes associated with glucose metabolism, uncovered their interactions, and deciphered the impact of these gut microbial interactions on the host glucose metabolism. Our findings may pave the way for the treatment of hyperglycemia from the perspective of gut microbial interactions.

## Introduction

Glucose and lipid metabolism are closely interrelated and play vital roles in maintaining the health status of the host [1, 2]. As has been reported, disordered glucose and lipid metabolism can lead to a series of metabolic diseases, such as overweight [3], obesity [4], diabetes [5], and hypertension [6]. In recent years, the incidence of major metabolic diseases caused by disordered glucose and lipid metabolism has increased enormously [7]. However, due to the complicated pathogenesis of diseases associated with glucose and lipid metabolism disorders, sefficient strategies for the treatment of these diseases are still lacking.

The gut microbiota is a complex microbial ecosystem that resides in the gastrointestinal (GI) tract of the host. It has been reported that the microbial composition of the gut is closely associated with the glucose and lipid metabolism of the host [8–10]. Many studies have revealed that the gut microbiota regulates glucose and lipid metabolism via different mechanisms. First, the gut microbiota affects the glucose metabolism of the host by reducing intestinal carbohydrates [11]. Second, the gut microbiota regulates glucose and lipid metabolism by synthesizing functional metabolites [12–15]. Third, the gut microbiota affects lipid absorption by shaping the intestinal immune system [16]. Last, the gut microbiota regulates lipid metabolism by sensing variations of external nutrition [17]. All of these studies have indicated that gut microbes play vital roles in glucose and lipid metabolism. Although many glucose and lipid metabolism–associated gut microbes have been identified, little relevant work has been done to decipher the impact of gut microbial interactions on glucose and lipid metabolism.

In the present study, we tried to identify the glucose- and lipid-metabolism–associated gut microbes by disturbing the gut microbial composition of high-fat diet (HFD)–fed mice. To fulfill this goal, a *Listeria monocytogenes* (Lmo)–derived bacteriocin Lmo2776 secretion module was constructed and integrated into the genome of *Escherichia coli* Nissle 1917 (EcN), forming the Lmo2776 secretion strain EcN-2776. Oral administration of this engineered strain to HFD-fed mice decreased blood glucose and increased serum triglyceride. In addition, the intestinal secretion of Lmo2776 adjusted the gut microbial composition of HFD-fed mice by decreasing the relative abundance of *Lactobacillus*, whereas increasing the relative abundance of *Faecalibaculum*. Mechanistic studies showed that Lmo2776 directly inhibited the growth of 3 *Lactobacillus* microbes. Among these microbes, *Ligilactobacillus murinus* was capable of secreting functional metabolites (xanthoxylin) to restrict the growth of *Faecalibaculum rodentium*. Oral administration of *F. rodentium* to the HFD-fed mice decreased the blood glucose level, a finding that might be attributable to the intestinal consumption of glucose by *F. rodentium*. Collectively, our study demonstrates the importance of gut microbial interactions in the metabolic activity of the host.

## Materials and methods

### Construction of the synthetic microbe EcN-2776

**Figure 1:**
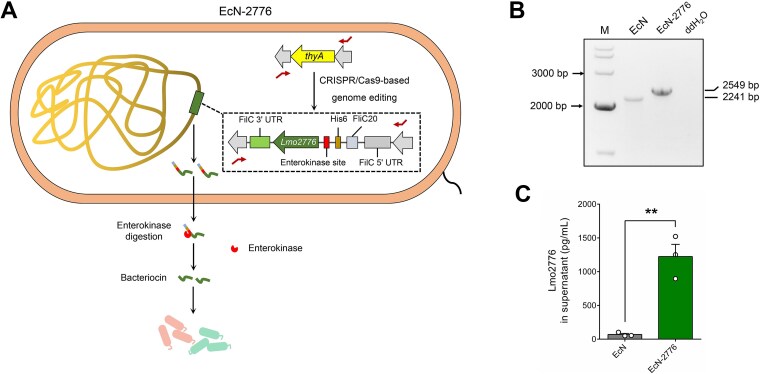
**Construction of EcN-derived synthetic microbe for the secretion of Lmo2776. A**. The construction of the Lmo2776-secreting strain (EcN-2776). **B**. Genotype verification of EcN-2776 by PCR amplification. Primers are labelled as bended arrows in (**A**). **C**. The measurement of His6-tagged Lmo2776 in the supernatants of EcN and EcN-2776. Data are presented as mean ± SEM. The statistical significance of the data between different groups was analyzed using the *t*-test (^*^^*^P < .01).

Lmo2776 is codon optimized according to the codon preference of *E. coli*. To facilitate the efficient secretion of Lmo2776 in EcN, the modified *E. coli* flagellar secretion apparatus [18] was used for the construction of an Lmo2776 secretion module, which contained the upstream untranslated DNA fragment of the gene *fliC* (FliC 5′ untranslated region [UTR]), the 20 N-terminal amino acids of FliC (FliC20), the His6 flag (His6), the enterokinase cleavage site (Asp-Asp-Asp-Asp-Lys [DDDDK]) (enterokinase site), the codon-optimized Lmo2776 (Lmo2776), and the downstream untranslated DNA fragment of the gene *fliC* (FliC 3′ UTR) (Fig. 1A). To promote the generation of untagged Lmo2776 in the intestine, the enterokinase cleavage site, which can be digested by enterokinase, was inserted between the His6 tag and Lmo2776.

To construct the Lmo2776 secretion module, the FliC 5′ UTR-FliC 20–amino acid fragment is polymerase chain reaction (PCR) amplified by using the primer pairs FliC 5′-s/FliC 20-a with the genome of *E. coli* MG1655 supplied as the template. The His6-enterokinase cleavage site-Lmo2776 fragment is PCR amplified by use of the primer pairs His6-s/Lmo2776-a with the codon-optimized Lmo2776 supplied as the template. The FliC 3′ UTR fragment is PCR amplified by use of the primer pairs FliC 3′-s/FliC 3′-a with the genome of *E. coli* MG1655 supplied as the template. Then, the Lmo2776 secretion module is constructed via overlap PCR by using the primer pairs FliC 5′-s/FliC 3′-a with the above-mentioned 3 fragments as templates.

To integrate the Lmo2776 secretion module into the genome of EcN, the secretion module is cloned into the *E. coli*-editing plasmid pTargetF, generating the plasmid pTargetF-Lmo2776. On the plasmid, the 500-bp upstream and downstream sequences of the *thyA* gene are used as homologous arms. The genome editing of EcN was performed according to previously reported procedures [19]. The Cas9 nuclease can target the *thyA* gene with the assistance of a single-guide RNA containing a 20-nucleotide guide sequence (CGCTCCTGTGACGTCTTCCT). After genome editing, we obtained the synthetic microbe EcN-2776, whose genotype was verified by PCR amplification and confirmed by DNA sequencing. The primers used for plasmid construction are listed in Supplementary Table S1.

### Measurement of His6-tagged Lmo2776

Both EcN and EcN-2776 were inoculated into 5 ml M9 medium and grown anaerobically (Electrotek AW500TG-D, UK) at 37°C until the optical density (OD)_600_ reached 0.8–1.0. Then, the grown cells are inoculated into 40 ml M9 medium and grown anaerobically at 37°C until OD_600_ reached 1.0. After centrifugation, the supernatants are collected and concentrated by using Amicon Ultra centrifugal filters (Millipore, UFC800324). The measurement of His6-tagged Lmo2776 was performed according to the His Tag enzyme-linked immunoassay (ELISA) detection kit instructions (GenScript, L00436).

### Animal experiments

Four-week-old male C57BL/6 J mice were purchased from Charles River Laboratories, China. The mice were maintained under pathogen-free conditions on a 12-hour light–dark cycle at a temperature of 24°C and humidity of 40%–60% and given free access to food and water.

To investigate the impact of EcN-2776 on the normal physiology of HFD (Research Diet, D12492)–fed mice, the mice were randomly divided into 4 groups: (1) normal diet (ND)–phosphate-buffered saline (PBS) (*n* = 11), ND-fed mice gavaged with PBS solutions; (2) HFD-PBS (*n* = 10), HFD-fed mice gavaged with PBS solutions; (3) HFD-EcN (*n* = 10), HFD-fed mice gavaged with 1 × 10^9^ colony forming units (CFUs) of viable EcN cells; and (4) HF–EcN-2776 (*n* = 11), HFD-fed mice gavaged with 1 × 10^9^ CFU viable EcN-2776 cells. Before the experiment, the mice were kept in ND- and HFD-fed condition for 60 days. Then, the mice in the 4 groups were gavaged with PBS solutions, EcN, and EcN-2776 3 times per week for 70 days. Fasting blood glucose was assayed by tail vein blood sampling using Accu-Chek Performa blood glucose meter test strips (Roche Diagnostics). The oral glucose tolerance test (OGTT) and insulin tolerance test (ITT) were performed as previously reported [20] at days 63 and 66. The areas under the curve (AUCs) of OGTT and ITT were calculated by using GraphPad. The serum concentrations of insulin, triglyceride, total cholesterol, interferon gamma (IFN-γ), interleukin 6 (IL-6), and lipopolysaccharide (LPS) were assayed at days 28 and 70 by using a mouse insulin ELISA (enzyme-linked immunosorbent assay) kit (Mlbio, ml001983-2), triglyceride assay kit (Jiancheng Bio, Nanjing, China, A110-1-1), total cholesterol assay kit (Jiancheng Bio, Nanjing, China, A111-1-1), and mouse IFN-γ, IL-6, and LPS ELISA kits (Mlbio, ml002277, ml063159, and ml037221-2), respectively. The routine blood and biochemical analyses were performed by using a chemistry analyzer (Catalyst One, 443 IDEXX).

To investigate the impact of *F. rodentium* on the blood glucose of HFD-fed mice, the mice were randomly divided into 2 groups: (1) the PBS group (*n* = 7), HFD-fed mice gavaged with PBS solutions; (2) the *F. rodentium* group (*n* = 7), HFD-fed mice gavaged with 1 × 10^9^ CFU viable cells of *F. rodentium*. After 1 week acclimation, the mice were treated with a broad-spectrum antibiotic cocktail (ampicillin 1 mg/ml, neomycin 1 mg/ml, vancomycin 0.5 mg/ml, and meropenem 0.5 mg/ml) in drinking water for 10 days. Then, the mice are gavaged with PBS solutions and *F. rodentium* 3 times per week. The body weight and fasting blood glucose were assayed at days 0, 7, and 20. The OGTT and ITT experiments were performed as previously reported [20] at days 17 and 19, respectively. The AUC of OGTT and ITT were calculated by using GraphPad. The serum concentration of triglyceride is assayed at day 20 according to the triglyceride assay kit instructions (Jiancheng Bio, Nanjing, China, A110-1-1).

### Ethics statement

The experimental protocol was approved by the Animal Care Committee at the Shanghai Institute of Biological Science, CAS/Center for Excellence in Brain Science and Intelligence Technology, Chinese Academy of Sciences (No. NA-041-2022-R1).

### 16S rRNA gene amplicon sequencing

Fecal genome extraction from mice in the ND-PBS, HFD-PBS, HFD-EcN, and HFD-EcN-2776 groups (day 70) was performed according to the standard protocol of the OMEGA Soil DNA Kit (Omega Bio-Tek, M5635-02). The quality of the genomic DNA was determined by agarose gel electrophoresis. The concentration of genomic DNA was assayed via a NanoDrop ND-2000 spectrophotometer (Thermo Scientific, USA). The V3–V4 region of the 16S rRNA gene was amplified by using the primers 338F (5′-ACTCCTACGGGAGGCAGCA-3′) and 806R (5′-GGACTACHVGGGTWTCTAAT-3′). The purified PCR products were paired-end sequenced by using the MiSeq PE300 platform (Illumina, San Diego, USA).

The clean reads were de-noised using DADA2 [21] and viewed as amplicon sequence variants (ASVs). Taxonomic assignment of ASVs was performed by using the Naive bayes consensus taxonomy classifier implemented in QIIME2 [22, 23] and the SILVA 16S rRNA database (v138). PICRUSt2 [24] was used for the prediction of gut microbial function.

Bioinformatic analysis of the gut microbiome was performed by using the Majorbio Cloud platform. The α-diversity indices were assayed using Mothur v1.30.2 [25]. Principal coordinate analysis based on Bray–Curtis distances was used to determine the similarity among the microbial communities in different samples. Venn diagrams were generated using the R package VennDiagram. The significantly abundant taxa of bacteria among different groups were identified by using the linear discriminant analysis effect size [26].

### Isolation of *Lactobacillus* microbes

The fecal samples of HFD-fed mice (100 mg) were dissolved in PBS solutions (1 ml) to acquire a homogenized solution. After serial dilution in PBS solutions, the fecal samples were spread onto the deMan Rogosa Sharpe (MRS) plate and grown anaerobically at 37°C for 24 h. Single-colony PCR was used to amplify the 16S rRNA. The *Lactobacillus* microbes were identified by using BLASTn with the sequenced 16S rRNA serving as the template.

### Interaction analysis between EcN/EcN-2776 and *Prevotella copri*

EcN, EcN-2776, and *P. copri* were inoculated into Yeast, Casitone, Fatty Acids, and Glucose (YCFAG) medium and grown anaerobically until the OD_600_ reached 1.0. The grown cells of EcN/EcN-2776 and *P. copri* were equally mixed and inoculated into YCFAG medium. The grown cells in the mixtures were collected at 10 h. The genomes of cell pellets were extracted by using Magen HiPure bacterial DNA kits (Magen, D3146-03). Then, a quantitative PCR assay was performed to measure the absolute abundance of *P. copri* and EcN/EcN-2776 in the mixtures. Considering that EcN/EcN-2776 and *P. copri* were equally mixed at the beginning of the experiment, the relative abundances of EcN/EcN-2776 and *P. copri* were considered to be the same at 0 h. The absolute abundances of EcN/EcN-2776 and *P. copri* at 10 h relative to those at 0 h were used to assay the relative abundance of EcN/EcN-2776 and *P. copri* in the mixtures at 10 h. The primers are listed in Supplementary Table 1.

### Interaction analysis between EcN/EcN-2776 and 3 *Lactobacillus* microbes

EcN and EcN-2776 were inoculated into Luria-Bertani (LB) medium and grown anaerobically until the OD_600_ reached 1.0. The 3 identified *Lactobacillus* microbes were inoculated into the MRS medium and grown anaerobically until OD_600_ reached 1.0. The grown cells of EcN/EcN-2776 and 3 *Lactobacillus* microbes were washed and dissolved in PBS solutions. The cells of EcN/EcN-2776 and 3 *Lactobacillus* microbes were equally mixed and anaerobically incubated at 37°C for 6 h. After centrifugation, the cell pellets were collected and used for DNA extraction with Magen HiPure bacterial DNA kits (Magen, D3146-03). A quantitative PCR assay was performed to determine the absolute abundance of EcN/EcN-2776 and 3 identified *Lactobacillus* microbes. The relative abundances of EcN/EcN-2776 and 3 identified *Lactobacillus* microbes in the mixtures were determined as mentioned above. The primers are listed in Supplementary Table S1.

### Lmo2776-based growth inhibition of *Lactobacillus* microbes

Lmo2776 was chemically synthesized as previously reported [27]. The 3 *Lactobacillus* microbes were inoculated into the MRS medium containing 3 μg/ml Lmo2776 (MRS-Lmo2776) or not (MRS) and grown anaerobically at 37°C for 6 h. The inhibitory effect of Lmo2776 was evaluated by assaying the OD_600_ of *Lactobacillus* microbes.

### Inhibitory impact of *Lactobacillus* microbes towards *F. rodentium*

The 3 identified *Lactobacillus* microbes were inoculated into Peptone Yeast Glucose (PYG) medium and grown anaerobically at 37°C until the OD_600_ reached 2.0. After centrifugation, the 3 *Lactobacillus* microbe*–*derived supernatants were collected. *F. rodentium* was inoculated into the PYG medium containing the supernatants of *Ligilactobacillus murinus*, *Limosilactobacillus reuteri*, and *Lactobacillus taiwanensis* or not and grown anaerobically at 37°C. The inhibitory effect of *Lactobacillus* microbes towards *F. rodentium* were determined by assaying the OD_600_ of *F. rodentium* at 0, 2, 4, 6, 8, and 10 h.

### Untargeted metabolomic analysis

The PYG medium and the supernatants of 3 *Lactobacillus* microbes were collected, filtered through the 0.22-μm membrane, and subjected to untargeted metabolomic analysis as reported previously [28, 29].

### Determining the absolute abundance of *L. murinus* by using quantitative PCR

The total amount of *L. murinus* DNA in stool samples was quantified using the following primers: LactoM-F (5′-TCGAACGAAACTTCTTTATCACC-3′) and LactoM-R (5′-CGTTCGCCACTCAACTCTTT-3′) [30]. Quantitative PCR was carried out on a LightCycler 480 II PCR system (Roche) with the following cycling conditions: 95°C for 5 min, 45 cycles at 95°C for 10 s, 60°C for 10 s, and 72°C for 10 s. A standard curve generated from a pMD18T vector containing the fragment that was amplified with the above-mentioned primers was used for normalization for each run of real-time PCR.

### Glucose-based growth promotion of *F. rodentium*

*F. rodentium* was inoculated into the PYG medium and grown anaerobically at 37°C until the OD_600_ reached 1.0. The grown cells are inoculated into the PYG medium (5% v/v) containing 0.5% and 1% glucose and grown anaerobically at 37°C. The OD_600_ was assayed at 0, 2, 4, and 6 h.

### Verification for the absence of contamination in the *in vitro* growth assays

To check the absence of contamination in the *in vitro* growth assays, the samples were collected before and after the experiment to acquire the cell pellets by centrifugation. Then, the genomes of cell pellets were extracted and used as templates to amplify the 16S rRNA for sequencing. The sequenced 16S rRNA served as the template by the use of BLASTn to ascertain that there were no contaminations in the *in vitro* growth assays.

### Quantification and statistical analysis

The statistical analysis data are presented as mean ± SEM. The statistical significance between 2 groups was analyzed using the Student *t*-test (unpaired, two-tailed). The statistical significance between more than 3 groups was analyzed using one-way ANOVA.

## Results

### Construction of the *Listeria* bacteriocin Lmo2776 secretion microbe

We tried to identify the glucose and lipid metabolism–associated gut microbes by adjusting the gut microbial composition of HFD-fed mice with a bacteriocin-targeting strategy. To fulfill this goal, the *L. monocytogenes*-derived bacteriocin Lmo2776 secretion module was constructed and integrated into the genome of EcN to displace the *thyA* gene, forming EcN-2776 (Fig. 1A). The genotype of EcN-2776 was verified by PCR amplification and confirmed by DNA sequencing (Fig. 1B). *In vitro* fermentation results indicated that EcN-2776 could secrete the His6-tagged Lmo2776 efficiently (Fig. 1C). To test whether the EcN-2776–secreted Lmo2776 was effective for targeting gut microbes, we examined the inhibitory effect of EcN-2776 on *P. copri* DSM 18205 (*P. copri*) (Supplementary Fig. S1A), whose growth had been reported to be inhibited by Lmo2776 [27]. Before the microbial interaction experiment, we compared the growth ability of *P. copri*, EcN, and EcN-2776 and found that EcN and EcN-2776 grew faster than *P. copri* from 0 to 6 h, whereas the OD_600_ of *P. copri* was far more than that of EcN and EcN-2776 from 8 to 22 h (Supplementary Fig. S1B). Then, the grown cells of *P. copri* and EcN/EcN-2776 were equally mixed and inoculated (1% v/v) into the YCFAG medium for growth. After incubation for 10 h, the grown cells were collected. Assisted with the *P. copri* and EcN-specific primers (Supplementary Fig. S1C), we performed a quantitative PCR assay to determine the absolute abundance of *P. copri* and EcN/EcN-2776 in the mixtures. Then, the absolute abundance of EcN/EcN-2776 or *P. copri* at 10 h relative to that of 0 h was used to assay the relative abundance of EcN/EcN-2776 and *P. copri* in the mixtures at 10 h. Our results indicated that EcN-2776 inhibited the growth of *P. copri*, whereas the growth of EcN was restricted by *P. copri* (Supplementary Figs. S1D and E). Thus, EcN-2776 was capable of secreting the functional bacteriocin Lmo2776.

**Figure 2:**
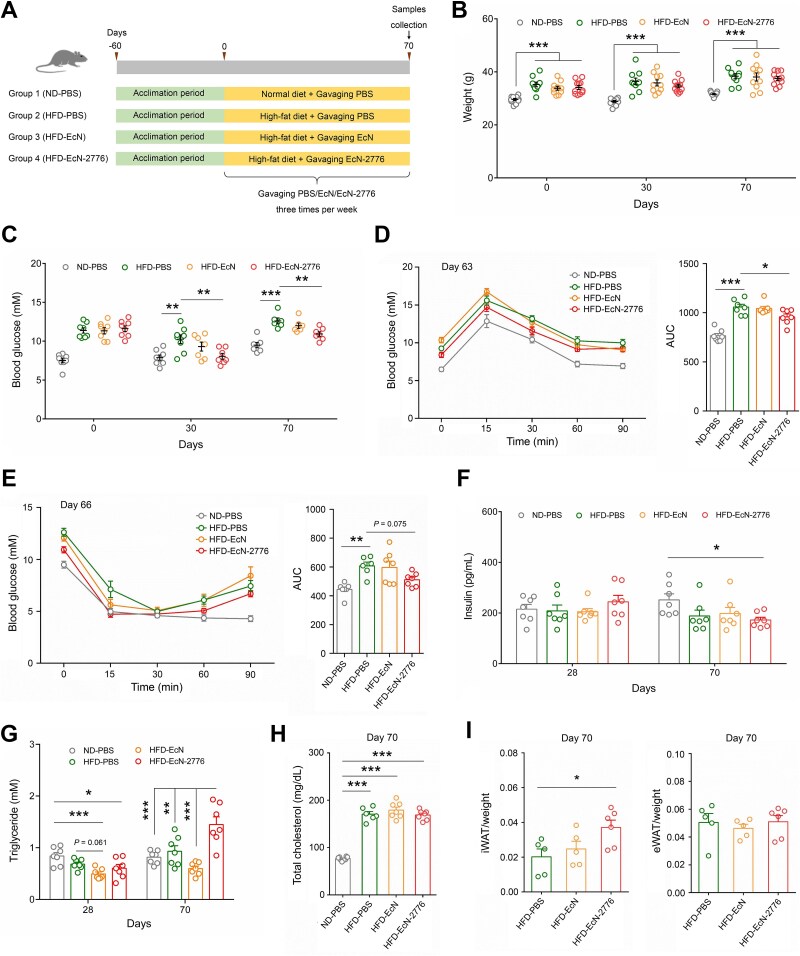
**Oral administration of EcN-2776 affects the glucose and lipid metabolism of HFD-fed mice. A**. Overview of animal experiments. Mice were gavaged with EcN and EcN-2776 3 times per week (1 × 10^9^ CFU per mouse). **B**. The weight of the 4 groups of mice at days 0, 30, and 70. **C**. Concentration of fasting blood glucose in the 4 groups at days 0, 30, and 70. **D**. Blood glucose profile and the AUC calculated during the OGTT (day 63). **E**. The blood glucose profile and AUC calculated during the ITT (day 66). **F**. Concentration of insulin in the 4 groups at days 28 and 70. **G**. Concentration of triglyceride in the 4 groups at days 28 and 70. **H**. The concentration of total cholesterol in the 4 groups at day 70. **I**. iWAT and eWAT weight ratio of the HFD-PBS, HFD-EcN, and HFD-EcN-2776 groups at day 70. Data are presented as mean ± SEM. The statistical significance between groups was analyzed by one-way ANOVA (^*^P < .05, ^*^^*^P < .01, ^*^^*^^*^P < .001).

### EcN-2776 administration affects the glucose and lipid metabolism of HFD-fed mice

To test the impact of Lmo2776 on glucose and lipid metabolism, both EcN (HFD-EcN group) and EcN-2776 (HFD–EcN-2776 group) were orally administered to the HFD-fed mice (Fig. 2A). The ND- and HFD-fed mice that were gavaged with PBS solution were used as controls (ND-PBS and HFD-PBS groups). The body weight showed no significant difference between the 4 groups of mice (Fig. 2B), whereas the fasting blood glucose of the HFD-EcN-2776 group was decreased at days 30 and 70 compared to the HFD-PBS group (Fig. 2C). OGTT analysis revealed that the mice in the HFD-EcN-2776 group showed a stronger glucose-lowering effect than that of the HFD-PBS and HFD-EcN groups (Fig. 2D). Similarly, the insulin resistance of the HFD-EcN-2776 group was decreased compared to that of the HFD-PBS group, indicating that Lmo2776 had a restorative effect on insulin resistance (Fig. 2E). Furthermore, the serum insulin and pancreas/body weight ratio of 3 HFD-fed groups of mice showed no significant differences (Fig. 2F; Supplementary Fig. S2A), indicating that the decreased blood glucose of the HFD–EcN-2776 group was not affected by insulin. Compared to the HFD-PBS group, serum triglyceride was decreased in the HFD-EcN group at days 28 and 70 whereas it was increased in the HFD–EcN-2776 group at day 70 (Fig. 2G). The total cholesterol of the 3 HFD-fed groups showed no significant difference at day 70 and was higher than that of the ND-PBS group (Fig. 2H). The inguinal white adipose tissue (iWAT)/body weight ratio of the HFD–EcN-2776 group was higher than that of the HFD-PBS group at day 70, but not higher for the epididymal white adipose tissue (eWAT)/body weight ratio (Fig. 2I). The brown adipose tissue (BAT)/body weight ratio was decreased in the HFD-EcN and HFD–EcN-2776 groups, whereas the liver/body weight ratio was decreased only in the HFD–EcN-2776 group (Supplementary Fig. S2B and C).

Hematoxylin and eosin (HE) staining was performed to characterize the histomorphological differences between the iWAT of the 3 HFD-fed groups of mice. The size of fat cells in the iWAT of the HFD-EcN-2776 group was larger than that of the HFD-PBS and HFD-EcN groups (Supplementary Fig. S3A). To ascertain the reason for the increase of iWAT in the HFD-EcN-2776 group, we quantified the expression of the genes related to glucose conversion and fatty acid storage in the iWAT of the 3 HFD-fed groups. For the glucose conversion–associated genes, the expression of the hexokinase 2 gene (*HK2*) was decreased in the HFD-EcN-2776 group compared with the HFD-PBS group, whereas the expressions of the acetyl-CoA carboxylase gene (*ACC*) and fatty acid synthase gene (*FASN*) showed no significant difference between these 2 groups of mice (Supplementary Fig. S3B). These results indicated that the increase of iWAT in the HFD-EcN-2776 group was not caused by glucose conversion. As for the fatty acid storage–associated gene, the expression of the diacylglycerol acyltransferase 2 type gene (*DGAT2*) was increased in the HFD-EcN-2776 group compared with the HFD-PBS group. Furthermore, the expression of the hormone-sensitive lipase gene (*HSL*) showed no significant difference between the HFD-PBS and HFD-EcN-2776 groups. Based on these results, we concluded that the increase of iWAT in the HFD-EcN-2776 group was caused by fatty acid storage (Supplementary Fig. S3C).

We checked the inflammatory state of 4 groups of mice and found that the concentrations of IFN-γ, IL-6, and LPS showed no significant differences between the 3 HFD-fed groups at days 28 and 70 (Supplementary Fig. S4A). The blood routine and blood chemistry test results showed that the concentration of blood urea nitrogen was decreased in the HFD-EcN-2776 group compared with the HFD-PBS group (Supplementary Fig. S4B). All these results revealed that the intestinal secretion of Lmo2776 affected the physiological activities of HFD-fed mice, especially those involving glucose and lipid metabolism.

### Intestinal secretion of Lmo2776 alters gut microbial composition of HFD-fed mice

**Figure 3:**
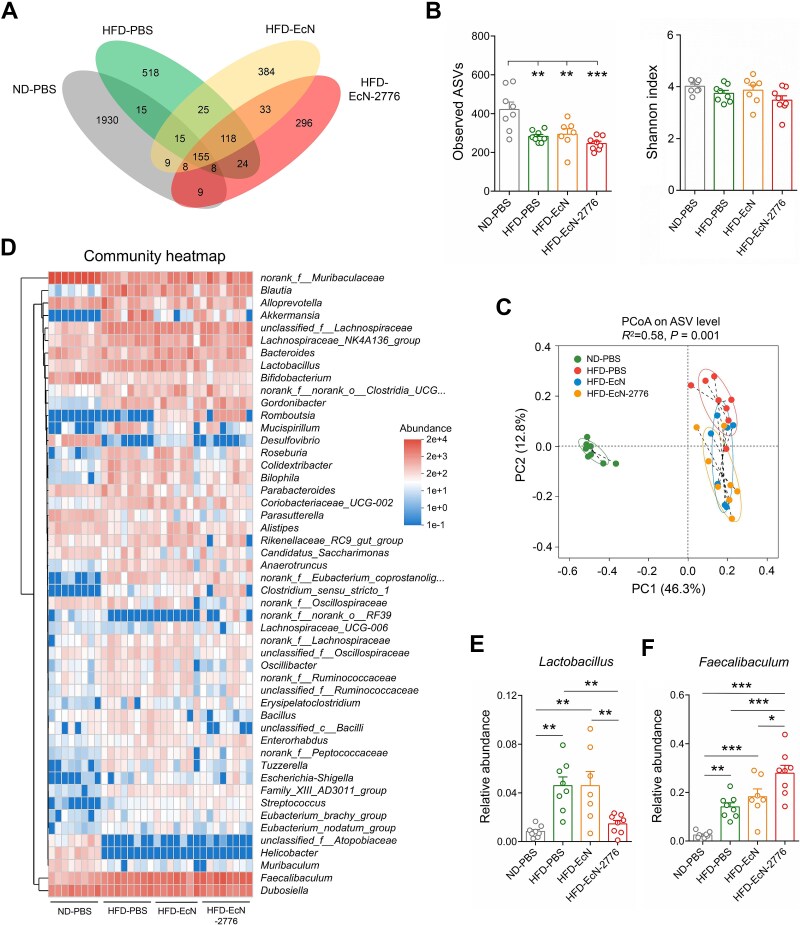
**Oral administration of EcN-2776 alters the gut microbial composition of HFD-fed mice. A**. Venn diagrams reveal the number of ASVs shared by the ND-PBS, HFD-PBS, HFD-EcN, and HFD–EcN-2776 groups. **B**. Richness (observed ASVs) and diversity (Shannon index) of gut microbiome between the 4 groups of mice. **C**. Principal coordinate analysis of Bray-Curtis distances based on the ASVs of the 4 groups. **D**. Community heatmap analysis of top 50 bacterial genera between the four mice groups. **E**. Relative abundance of *Lactobacillus* in the 4 groups. **F**. Relative abundance of *Faecalibaculum* in the 4 groups. Data are presented as the mean ± SEM. The statistical significance between different groups was analyzed by one-way ANOVA (^*^P < .05, ^*^^*^P < .01, ^*^^*^^*^P < .001).

To determine the impact of EcN-2776 administration on the gut microbiome, the fecal samples of 4 groups of mice were collected for 16S rRNA gene amplicon sequencing. The number of 16S rRNA gene amplicon sequencing procedures performed from each sample were rarefied to 39 986, yielding an average Good’s coverage over 99% (Supplementary Fig. S5A). A Venn diagram showed that 155 amplicon sequence variants (ASVs) were shared by the 4 groups of mice, whereas 1930, 518, 384, and 296 ASVs exclusively belonged to the ND-PBS, HFD-PBS, HFD-EcN, and HFD-EcN-2776 groups (Fig. 3A). Although the microbial diversity (Shannon index) showed no obvious difference between the 4 groups, the microbial richness (observed ASVs) of the 3 HFD-fed groups was lower than that of the ND-PBS group (Fig. 3B). The gut microbial composition of the 3 groups of HFD-fed mice were quite different from that of the ND-PBS group (Fig. 3C). Furthermore, the gut microbial compositions of the HFD-EcN and HFD-EcN-2776 groups were different from the those of the HFD-PBS group (Fig. 3C). As for the distinguishing bacterial taxa between the 4 groups of mice, results of linear discriminant analysis effect size analysis revealed that *Bifidobacterium*, *Desulfovibrio*, and *Parasutterella* were abundant in the ND-PBS group; *Blautia*, *Akkermansia*, *Lactobacillus*, and *Mucispirillum* were enriched in the HFD-PBS group; *Bacteroides*, *Roseburia*, and *Alistipes* were abundant in the HFD-EcN group; and *Faecalibaculum*, *Gordonibacter*, and *Romboutsia* were enriched in the HFD-EcN-2776 group (Supplementary Fig. S5B).

To further explore the gut microbial differences between the 4 groups of mice, the relative abundance of microbiota at the phylum, family, and genus levels were analyzed. At the phylum level, *Verrucomicrobiota* was decreased in HFD-EcN and HFD-EcN-2776 groups compared to the HFD-PBS group (Supplementary Fig. S5C). At the family level, *Akkermansiaceae* was decreased in HFD-EcN and HFD-EcN-2776 groups compared with the HFD-PBS group; *Bacillaceae*, *Lactobacillaceae*, and unclassified_c_*Bacilli* were increased in the HFD-PBS and HFD-EcN groups and decreased in the HFD-EcN-2776 group; *Streptococcaceae* and *Peptostreptococcaceae* were abundant in HFD-EcN-2776 group (Supplementary Fig. S5D). At the genus level, the top 50 genera of the 3 groups of mice were used to perform a community heatmap analysis (Fig. 3D). Among these genera, *Lactobacillus* was decreased in the HFD–EcN-2776 group compared with the HFD-PBS group, whereas *Faecalibaculum* was increased (Fig. 3E and F). In addition, compared to the HFD-PBS group, *Akkermansia* was decreased in the HFD-EcN and HFD-EcN-2776 groups, whereas *Alistipes* was increased in the HFD-EcN group (Supplementary Fig. S5E).

The gut microbial function of the 4 groups of mice were predicted with PICRUSt2. Specifically, the Lg-transformed abundance of 5 enzymes (HMGS, HMGR, MvaK1, MvaD, and MvaK2) in the mevalonate pathway were decreased in the HFD-EcN-2776 group compared with the HFD-PBS and HFD-EcN groups (Supplementary Fig. S6A and B). Considering that *Lactobacillus* was also decreased in the HFD-EcN-2776 group (Fig. 3E), we performed the linear regression analysis to test the correlation of *Lactobacillus* with the above-mentioned 5 enzymes. The relative abundance of *Lactobacillus* was positively correlated with the Lg-transformed abundance of HMGS, HMGR, MvaK1, MvaD, and MvaK2 (Supplementary Fig. S6C–G). As previously reported, the mevalonate pathway was found to be responsible for the production of isoprenoids [31, 32]; therefore, the decrease in *Lactobacillus* may have restricted the intestinal synthesis of isoprenoids in the HFD–EcN-2776 group. This assumption was further supported by the existence of mevalonate pathway genes in various *Lactobacillus* species (Supplementary Fig. S7).

All of these results indicated that the EcN-2776–based intestinal secretion of Lmo2776 altered the gut microbial composition and function of HFD-fed mice.

### Growth of *Lactobacillus* microbes is inhibited by Lmo2776

**Figure 4:**
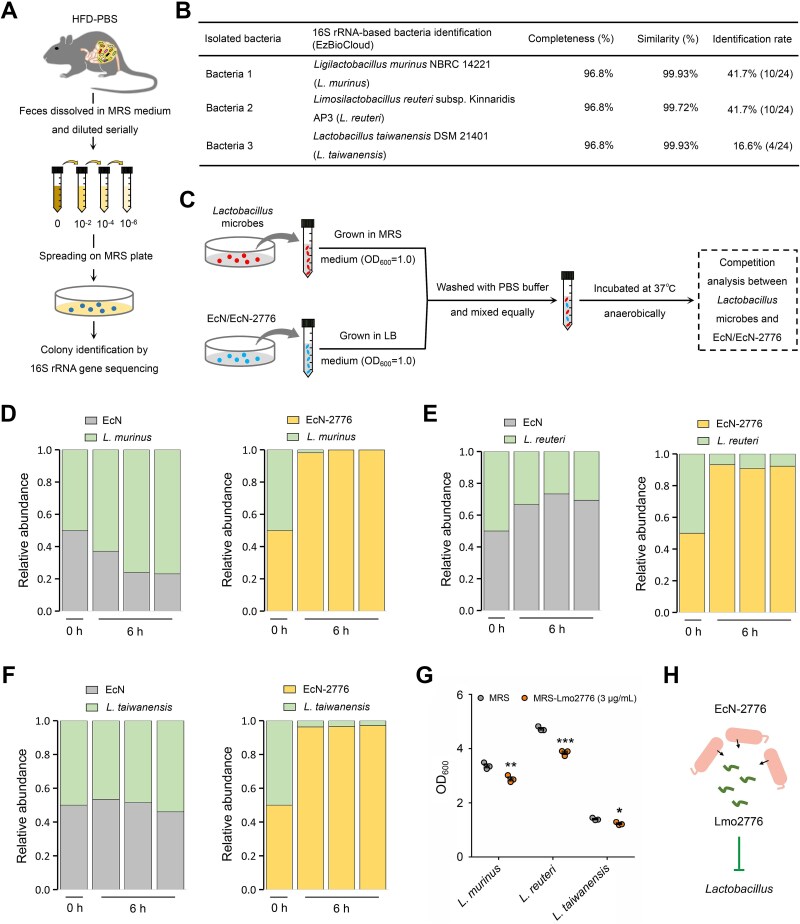
**Lmo2776 inhibits the growth of *Lactobacillus* microbes. A**. Schematic diagram showing the strategy for the isolation of *Lactobacillus* microbes from the feces of HFD-fed mice. **B**. Basic information of 3 isolated *Lactobacillus* microbes. **C**. Overview of the microbial interaction experiments. The collected *Lactobacillus* microbes and EcN/EcN-2776 were equally mixed in the PBS buffer and incubated anaerobically at 37°C for 6 h. **D**. Relative abundance of EcN/EcN-2776 and *L. murinus* in the mixtures at 0 and 6 h. **E**. Relative abundance of EcN/EcN-2776 and *L. reuteri* in the mixtures at 0 and 6 h. **F**. Relative abundance of EcN/EcN-2776 and *L. taiwanensis* in the mixtures at 0 and 6 h. **G**. The impact of Lmo2776 on the growth of 3 *lactobacillus* microbes at 6 h. **H**. Schematic diagram illustrating the growth of *Lactobacillus* microbes are inhibited by the EcN-2776-derived Lmo2776. Data are presented as mean ± SEM. The statistical significance of the data between the 2 groups was analyzed using the *t*-test (^*^P < .05, ^*^^*^P < .01, ^*^^*^^*^P < .001).

Given that the relative abundance of *Lactobacillus* was decreased in the HFD-EcN-2776 group (Fig. 3E), we wondered whether the growth of *Lactobacillus* microbes was inhibited by Lmo2776. To confirm this hypothesis, we isolated 3 *Lactobacillus* microbes (*Ligilactobacillus murinus* NBRC 14221 [*L. murinus*], *Limosilactobacillus reuteri* subsp. Kinnaridis AP3 [*L. reuteri*], and *Lactobacillus taiwanensis* DSM 21401 [*L. taiwanensis*]) from the feces of HFD-fed mice (Fig. 4A and B). Then, the inhibitory effect of Lmo2776 towards these 3 *Lactobacillus* microbes was verified by mixing the PBS-washed cells of *Lactobacillus* microbes with EcN/EcN-2776 (Fig. 4C). After incubation at 37°C for 6 h, the cells were collected and subjected to quantitative PCR assay by using EcN- and *Lactobacillus* microbe-specific primers (Supplementary Fig. S8) to determine the absolute abundance of *Lactobacillus* microbes and EcN/EcN-2776 in the mixtures. The absolute abundance of *Lactobacillus* microbes or EcN/EcN-2776 at 6 h relative to of the abundance at 0 h was used to assay the relative abundance of *Lactobacillus* microbes and EcN/EcN-2776 in the mixtures at 6 h. EcN-2776 exerted potent inhibitory effects on *L. murinus*, *L. reuteri*, and *L. taiwanensis* (Fig. 4D–F). Lmo2776 addition inhibited the growth of 3 *Lactobacillus* microbes (Fig. 4G). Thus, our results indicated that the growth of *Lactobacillus* microbes was inhibited by Lmo2776 (Fig. 4H).

### *L. murinus* inhibits the growth of *F. rodentium*

**Figure 5:**
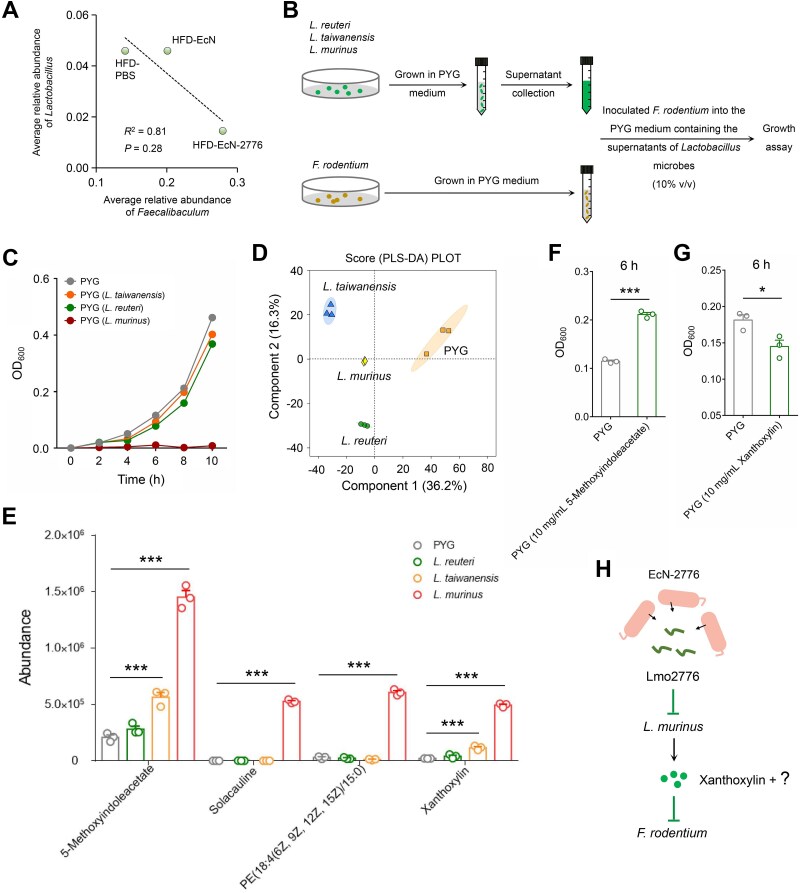
***L. murinus* inhibits the growth of *F. rodentium* by secreting functional metabolites. A**. Correlation between the average relative abundance of *Lactobacillus* and *Faecalibaculum*. **B**. Strategy used to investigate the impact of *Lactobacillus* microbes on the growth of *F. rodentium*. **C**. The impact of supernatants from 3 *Lactobacillus* microbes on the growth of *F. rodentium*. **D**. Partial least-squares discriminant analysis of the difference in metabolites between the PYG medium and the supernatants of 3 *Lactobacillus* microbes. **E**. Abundance of 4 metabolites that were enriched in the supernatant of *L. murinus*. **F and G**. Impact of 5-methoxyindoleacetate and xanthoxylin on the growth of *F. rodentium*. **H**. Schematic diagram illustrating the relationships between Lmo2776, *L. murinus*, and *F. rodentium*. Data are presented as mean ± SEM. The statistical significance between different groups was analyzed by one-way ANOVA (^*^P < .05, ^*^^*^^*^P < .001).

Our previous results revealed that the increased abundance of *Faecalibaculum* was accompanied by the decrease of *Lactobacillus* (Fig. 3E and F), indicating that *Faecalibaculum* microbes might be inhibited by *Lactobacillus* microbes. To test this hypothesis, we performed linear regression analysis to investigate the correlation between *Lactobacillus* and *Faecalibaculum* in 3 HFD-fed groups of mice. The average relative abundance of *Lactobacillus* was negatively correlated with *Faecalibaculum* (Fig. 5A). Next, we investigated the inhibitory impact of 3 identified *Lactobacillus* microbes on the growth of *F. rodentium* by inoculating *F. rodentium* into the PYG medium containing the supernatants of *L. murinus*, *L. reuteri*, and *L. taiwanensis* (Fig. 5B). The growth of *F. rodentium* was inhibited by the supernatant of *L. murinus* but not by the supernatants of *L. reuteri* and *L. taiwanensis* (Fig. 5C), indicating that the specific metabolites exclusively secreted by *L. murinus* were responsible for the inhibition of *F. rodentium*. To identify these metabolites, we performed an untargeted metabonomic analysis of the PYG medium (PYG group) and the supernatants of *L. murinus*, *L. reuteri,* and *L. taiwanensis*. Partial least-squares discriminant analysis indicated that the metabolites of 3 *Lactobacillus* microbes were quite different from that of the PYG group (Fig. 5D). Next, we screened the metabolites that exclusively enriched in the *L. murinus* group and found that 5-methoxyindoleacetate, solacauline, PE(18:4(6Z, 9Z, 12Z, 15Z)/15:0)), and xanthoxylin were abundant in the supernatant of *L. murinus* (Fig. 5E). Considering that solacauline and PE(18:4(6Z, 9Z, 12Z, 15Z)/15:0)) were two metabolites that unable to be purchased or acquired, we tested the impact of 5-methoxyindoleacetate and xanthoxylin on the growth of *F. rodentium* by inoculating *F. rodentium* into the medium containing 10 mg/ml 5-methoxyindoleacetate and xanthoxylin or not (PYG group). The growth of *F. rodentium* was promoted by 5-methoxyindoleacetate at 6 h, whereas inhibited by xanthoxylin (Fig. 5F and G). Based on these results, we concluded that *L. murinus* could inhibit the growth of *F. rodentium* by secreting functional metabolites, such as xanthoxylin (Fig. 5H).

To determine whether the isolated *L. murinus* was the same species in the gut microbiome of four mice groups in Fig. 3E, we assayed the absolute abundance of *L. murinus* (copies/g feces) by using the quantitative PCR assay. *L. murinus* was detected in the gut microbiome of ND- and HFD-fed mice, whose abundance was decreased in the HFD-EcN-2776 group as compared to the HFD-PBS and HFD-EcN groups (Supplementary Fig. S9). Thus, *L. murinus* was the same species in the gut microbiome of ND-PBS, HFD-PBS, HFD-EcN, and HFD-EcN-2776 groups.

### *F. rodentium* administration decreases the blood glucose of HFD-fed mice

**Figure 6:**
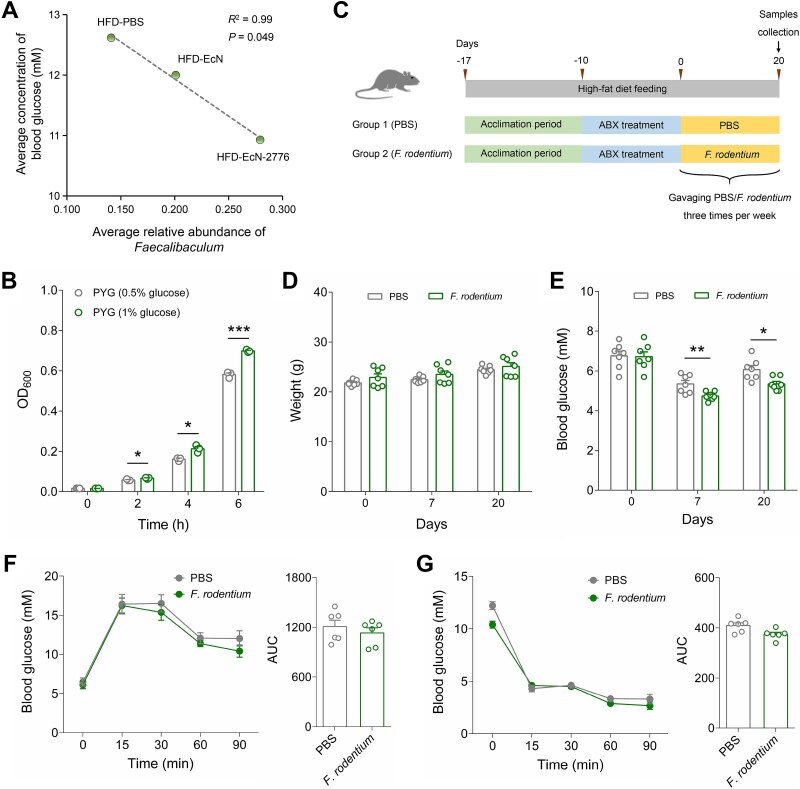
**Oral administration of *F. rodentium* exerts antihyperglycemic effects in HFD-fed mice. A**. Linear regression analysis for the correlation between the average blood glucose concentration and the average relative abundance of *Faecalibaculum* in 3 groups of HFD-fed mice. **B**. The growth of *F. rodentium* in the medium containing different glucose concentrations. **C**. Overview of animal experiments. Mice were gavaged with *F. rodentium* 3 times per week (1 × 10^9^ CFU per mouse). PBS, mice gavaged with PBS. *F. rodentium*, the mice gavaged with *F. Rodentium*. **D**. Weight of mice in PBS and *F. rodentium* groups at days 0, 7, and 20. **E**. Concentration of fasting blood glucose in PBS and *F. rodentium* groups at days 0, 7, and 20. **F**. Blood glucose profile and AUC calculated during the OGTT. **G**. Blood glucose profile and AUC calculated during the ITT. Data are presented as mean ± SEM. Statistical significance of the data between two groups was analyzed using the *t*-test (^*^P < .05, ^*^^*^P < .01, ^*^^*^^*^P < .001).

We performed linear regression analysis to investigate the correlation between *Faecalibaculum* and blood glucose. The average relative abundance of *Faecalibaculum* was negatively correlated with the average concentration of blood glucose (Fig. 6A). By assaying the growth of *F. rodentium* in the medium containing different glucose concentrations, we found that *F. rodentium* exerted a growth advantage in the high glucose concentration-containing medium (Fig. 6B). This result indicated that the enriched *Faecalibaculum* microbes in the HFD-EcN-2776 group might be responsible for its decreased blood glucose by consuming the intestinal glucose directly. To verify this hypothesis, *F. rodentium* was orally administered to the ABX-treated HFD-fed mice (*F. rodentium* group) (Fig. 6C). The mice that were gavaged with PBS solutions were used as controls (PBS group). The clearance of gut microbes in the ABX-treated groups was verified by 16S rRNA-based quantitative PCR assay at day 0 (Fig. 6C; Supplementary Fig. S10). The body weight of the two mice groups showed no significant differences at days 0, 7, and 20, whereas the fasting blood glucose of *F. rodentium* group was lower than that of PBS group at days 7 and 20 (Fig. 6D and E). OGTT analysis revealed that the *F. rodentium* group showed no significant glucose-lowering effect than that of PBS group (Fig. 6F). As for ITT, no significant insulin tolerance was observed in the *F. rodentium* group (Fig. 6G). To investigate the correlation between *Faecalibaculum* microbes and lipid metabolism, we performed the linear regression analysis and found that there was no significant correlation between the average relative abundance of *Faecalibaculum* and the average concentration of serum triglyceride (Supplementary Fig. S11A). Furthermore, the serum triglyceride concentration of the *F. rodentium* group was comparable to that of the PBS group at day 20 (Supplementary Fig. S11B), indicating that the *Faecalibaculum* microbes were not involved in lipid metabolism.

**Figure 7:**
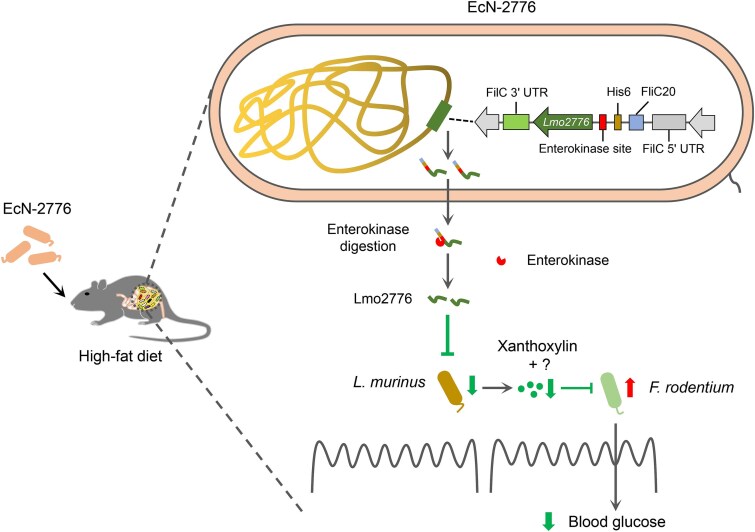
Schematic diagram illustrating the impact of EcN-2776 administration on the gut microbial interaction and blood glucose levels of HFD-fed mice.

Collectively, our results revealed that oral administration of EcN-2776 could restrict the growth of *L. murinus* by secreting Lmo2776, thus alleviating its inhibitory effect towards *F. rodentium*. The enrichment of *F. rodentium* might contribute to the antihyperglycemic effect of HFD-fed mice by consuming intestinal glucose directly (Fig. 7).

## Discussion

Gut microbiota acted as a critical factor that involved in the progression of various diseases, which were caused by the disordered glucose and lipid metabolism [33, 34]. Our study applied the bacteriocin-targeting strategy to adjust the gut microbial composition of HFD-fed mice and revealed a *Lactobacillus*-*Faecalibaculum* interaction that closely associated with the glucose metabolism of HFD-fed mice.

Bacteriocins were potent antimicrobial peptides that produced by a broad variety of bacteria [35, 36]. It had been reported that the *Listeria* bacteriocin Lmo2776 could target *Prevotella* microbes and reduce their abundance [27]. This conclusion was further supported by our study (Supplementary Fig. S1). Besides, apart from targeting *Prevotella* microbes, our study revealed that Lmo2776 inhibited the growth of 3 *Lactobacillus* microbes that isolated from the feces of HFD-fed mice (Fig. 4D–G). Based on these results, we concluded that Lmo2776 exerted antibacterial activity towards the microbes that belong to different genera.

The EcN-2776-based intestinal secretion of Lmo2776 could not only affect the glucose and lipid metabolism of HFD-fed mice, but also adjust their gut microbial composition (Figs. 2 and 3). Although our results demonstrated that the Lmo2776-induced enrichment of *F. rodentium* was the causal agent for the antihyperglycemic effect of EcN-2776-gavaged HFD-fed mice (Fig. 6E), oral administration of this bacteria to HFD-fed mice showed no significant impact on the serum triglyceride concentration (Supplementary Fig. S11B).

Apart from *Lactobacillus* and *Faecalibaculum*, Lmo2776 intervention could also affect the abundance of microbes belonging to *Bacillaceae* and unclassified_c_*Bacilli*, (Supplementary Fig. S5D). As reported, some of the *Bacillaceae* microbes could relieve the disordered lipid metabolism of host by secreting functional metabolites [37]. Thus, we assumed that the decreased abundance of *Bacillaceae* and unclassified_c_*Bacilli* in HFD-EcN-2776 group might contribute to the increased serum concentration of triglyceride. Besides, the gut microbe-derived or -modified metabolites played vital roles in the lipid metabolism of host [15, 38]. Thus, we will identify the functional metabolites that were responsible for the hyperlipidemia phenotype of EcN-2776-gavaged mice by performing the metabolomic analyses of fecal and blood samples in future studies.

Consistent with the hyperlipidemia phenotype, the iWAT/body weight ratio of HFD–EcN-2776 group was higher than that of the HFD-PBS group, whereas the eWAT/body weight ratio showed no difference between these 2 groups (Fig. 2I). Therefore, we assumed that iWAT might be the key tissue for the storage of excess triglycerides. In line with this assumption, the transgenic mice that expressing the cyclooxygenase-2 (COX-2) encoding gene (*Ptgs2*) in adipocytes selectively reduced the iWAT mass and fat cell size, but not for eWAT [39]. Considering the significant differences between iWAT and eWAT [40, 41], it was essential to decipher the specific mechanism for the storage of fat in iWAT and eWAT.

Oral administration of EcN and EcN-2776 decreased the relative abundance of *Akkermansia* (Supplementary Fig. S5E), indicating that EcN could inhibit the intestinal colonization of *Akkermansia* microbes. Compared to the HFD-PBS and HFD-EcN-2776 groups, the relative abundance of *Alistipes* was higher in the HFD-EcN group (Supplementary Fig. S5E), suggesting that the EcN administration-induced enrichment of *Alistipes* could be compensated for by the intestinal secretion of Lmo2776 in the HFD-EcN-2776 group. Thus, Lmo2776 might be capable of targeting *Alistipes* microbes, a finding that requires further verification.

It had been reported that the relative abundance of *Lactobacillus* showed opposite trends compared with *Faecalibaculum* under different conditions [42–44]. Consistent with these results, our study revealed that *L. murinus* could inhibit the growth of *F. rodentium* by secreting functional metabolites (Fig. 5C). Differently, the *L. reuteri-* and *L. taiwanensis*-derived metabolites showed no significant impact on the growth of *F. rodentium* (Fig. 5c). Therefore, in order to identify the *L. murinus*-derived metabolites for the inhibition of *F. rodentium*, the supernatants that collected from the grown cells of *L. murinus*, *L. reuteri*, and *L. taiwanensis* were subjected to comparative metabolomic analyses. By focusing on the metabolites that exclusively enriched in the supernatant of *L. murinus*, our results indicated that xanthoxylin, a metabolite that exhibited antifungal and antispasmodic activities, belonged to one of the functional metabolites for the inhibition of *F. rodentium*.

*F. rodentium* had been reported to be involved in various physiological activities of host. For example, it could promote the epithelial proliferation and turnover of mice by dampening the production of retinoic acid [45]; Oral administration of *F. rodentium* to the Apc^Min/+^ or azoxymethane- and dextran sulfate sodium-treated mice inhibited the growth of tumor [46]; *F. rodentium* could secrete functional factors to decrease the concentration of secretory IgA in feces, thus modulating the gut microbial composition [47]. It had been reported that feeding the high sugar-containing western style diet to mice could increase the intestinal abundance of *F. rodentium* [16]. Consistent with this result, our study showed that *F. rodentium* exerted growth advantage in the high glucose-containing medium and oral administration of *F. rodentium* could decrease the fasting blood glucose of ABX-treated HFD-fed mice (Fig. 6B and E). All these results suggested that *F. rodentium* might affect the glucose metabolism of host by consuming the intestinal glucose directly.

## Conclusion

Our study identified the glucose metabolism-associated gut microbes, uncovered their interactions, and deciphered the impact of gut microbial interaction on host glucose metabolism. Mechanistically, the intestinal secretion of Lmo2776 facilitated the enrichment of *F. rodentium* in the intestine of HFD-fed mice by restricting the growth of *L. murinus*, which could inhibit the growth of *F. rodentium* by secreting functional metabolites, such as xanthoxylin. Furthermore, oral administration of *F. rodentium* exerted antihyperglycemic effect to the HFD-fed mice, which might be achieved by the intestinal consumption of glucose by *F. rodentium*. All of these findings demonstrated the close association between gut microbial interactions and host metabolism and shined lights on treating hyperglycemia from the perspective of gut microbial interactions.

## Supplementary Material

25-2-8_Supplementary_information_wraf028

## Data Availability

The 16S rRNA gene amplicon sequencing data used in this study are available in the Sequence Read Archive of NCBI with Accession No. PRJNA1195686.
