# Supplementary material for: Murine gut microbial interactions exert antihyperglycemic effects
Source: ISME J. 2025 Feb 17;19(1):wraf028. doi: 10.1093/ismejo/wraf028 (PMC11896791; doi:10.1093/ismejo/wraf028)
Supplement: 25-2-8_Supplementary_information_wraf028 [file 25-2-8_supplementary_information_wraf028.docx]

**Supplementary information for:**

**Murine gut microbial interactions exert anti-hyperglycemic effects**

Liying Guo,^1,2^ Libing Xu,^1,2^ Yanhong Nie,^3,4,5^ Lu Liu,^3,4,5^ Zongping Liu,^1,2^ and Yunpeng Yang^1,2,3,4,*^

Running title: Gut microbes co-regulate hyperglycemia

^1^Jiangsu Co-innovation Center for Prevention and Control of Important Animal Infectious Diseases and Zoonoses, College of Veterinary Medicine, Yangzhou University, Yangzhou, 225009, China

^2^Institute of Comparative Medicine, Yangzhou University, Yangzhou, 225009, China

^3^Shanghai Center for Brain Science and Brain-Inspired Technology, Shanghai, 201602, China

^4^Institute of Neuroscience, CAS Key Laboratory of Primate Neurobiology, State Key Laboratory of Neuroscience, CAS Center for Excellence in Brain Science and Intelligence Technology, Chinese Academy of Sciences, Shanghai, 200031, China

^5^Key Laboratory of Genetic Evolution & Animal Models, Kunming Institute of Zoology, Chinese Academy of Sciences, Kunming, Yunnan 650223, China

^*^Corresponding author: Yunpeng Yang, College of Veterinary Medicine, Yangzhou University, 48 East Wenhui Road, Yangzhou, 225009 Jiangsu Province, People’s

Republic of China. Email: [ypyang@yzu.edu.cn](mailto:ypyang@yzu.edu.cn,).


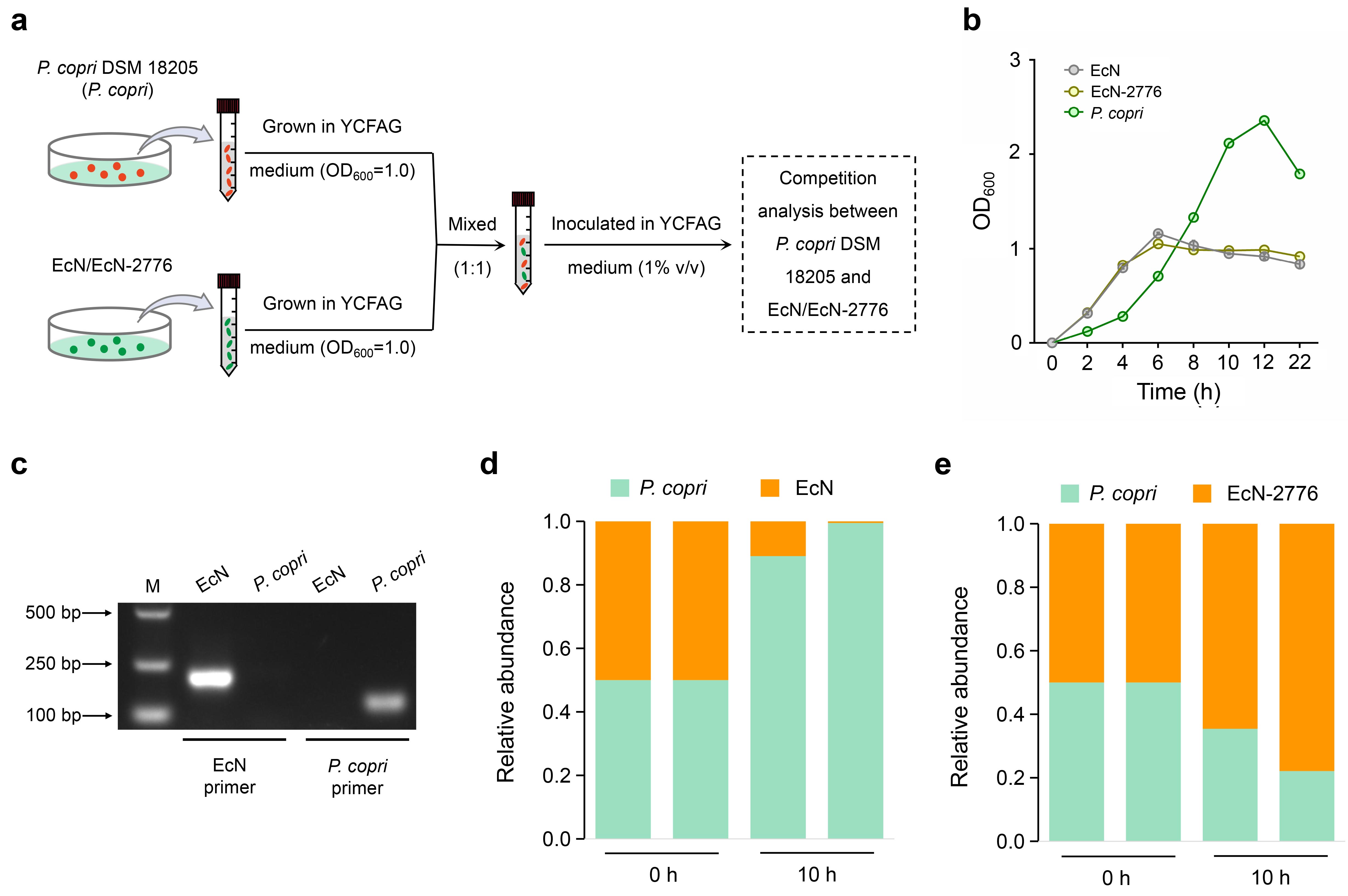


**Figure S1.** **EcN-2776 inhibits the growth of *P. copri.* a** Schematic diagram showing the strategy used for bacterial interaction analysis. **b** The growth curve of EcN, EcN-2776, and *P. copri* in YCFAG medium. **c** PCR-based verification of specific primers for EcN and *P. copri*. **d** The relative abundance of EcN and *P. copri* in the mixtures at 0 and 10 h. **e** The relative abundance of EcN-2776 and *P. copri* in the mixtures at 0 and 10 h.


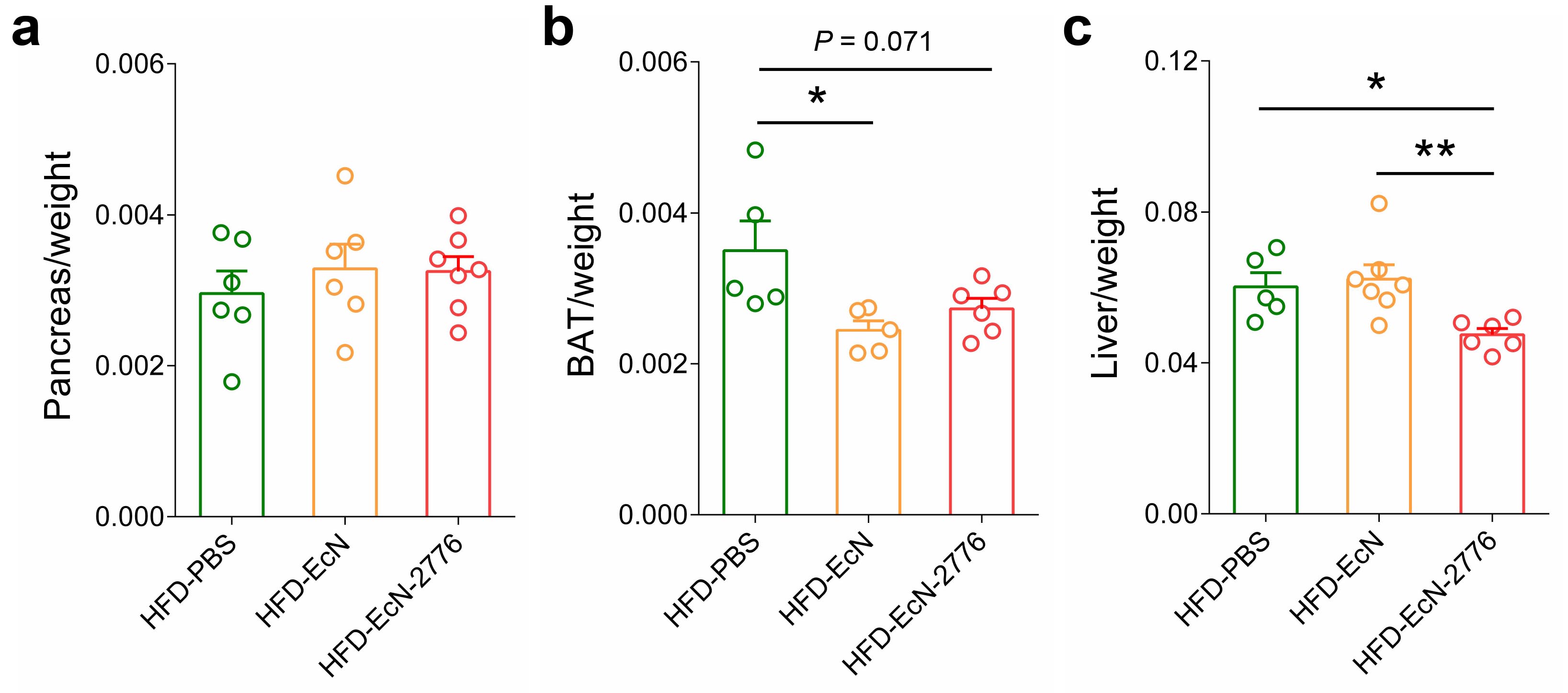


**Figure S2.** **Comparing the organ body weight ratio between the** **HFD-PBS, HFD-EcN, and HFD-EcN-2776 groups.** **a** The pancreas body weight ratio of three mice groups at day 70. **b** The brown adipose tissue (BAT) body weight ratio of three mice groups at day 70. **c** The liver body weight ratio of three mice groups at day 70. Data is presented in mean ± SEM. The statistical significance between different groups is analyzed by one-way ANOVA (**P* < 0.05, ***P* < 0.01).


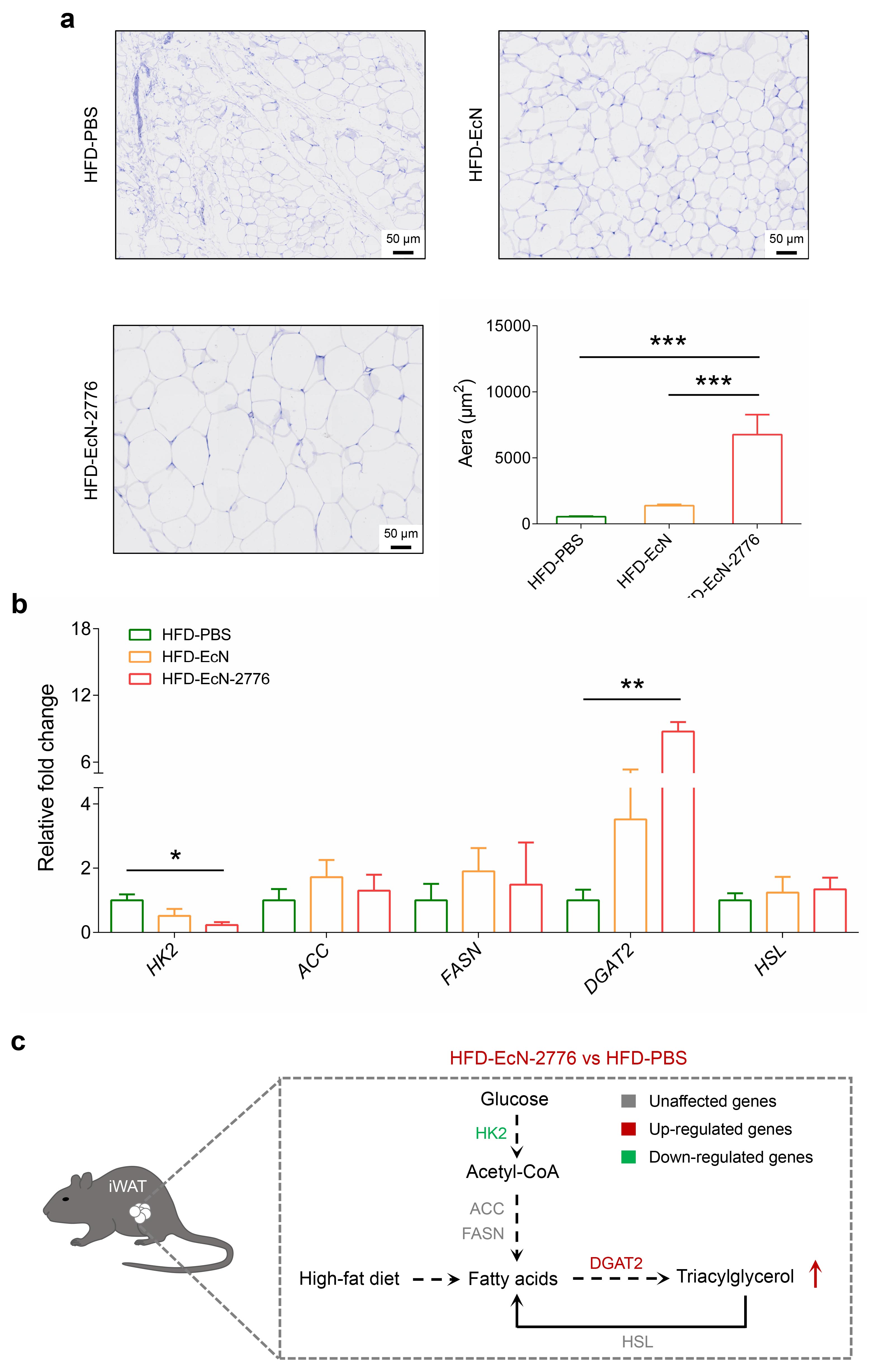


**Figure S3.** **Hematoxylin and eosin (HE) staining and gene expression analysis of** **iWAT in three HFD-fed mice groups.** **a** HE staining showing the histomorphological differences in the iWAT of three HFD-fed mice groups. **b** Comparing the expression of *HK2*, *ACC*, *FASN*, *DGAT2*, and *HSL* in the iWAT of three HFD-fed mice groups. **c** Schematic diagram illustrating the storage of fatty acids in the iWAT of HFD-EcN-2776 group. The up-regulated, down-regulated, and unaffected genes were labelled as red, green, and gray respectively. Data is presented in mean ± SEM. The statistical significance between different groups is analyzed by one-way ANOVA (**P* < 0.05, ***P* < 0.01, ****P* < 0.001).


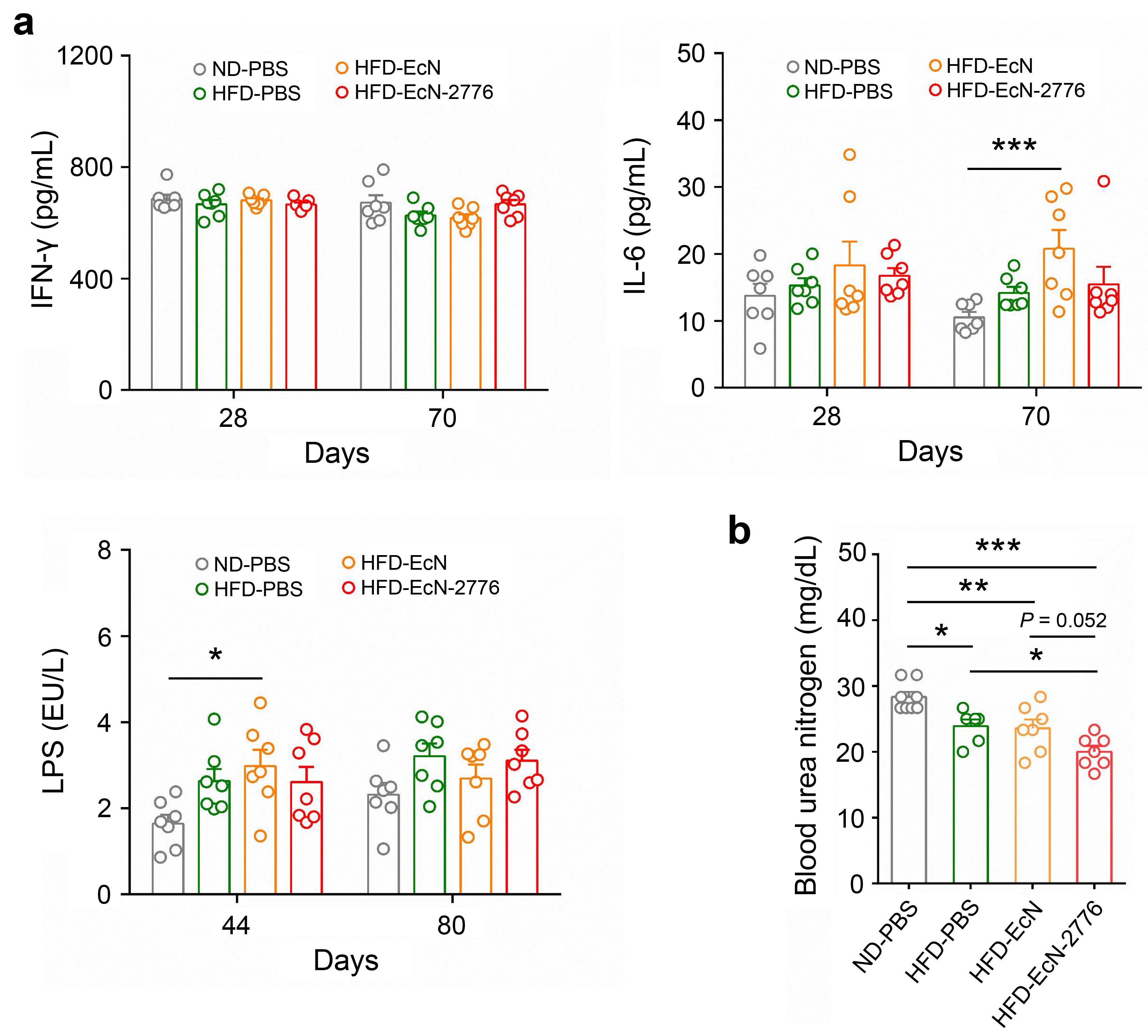


**Figure S4.** **Comparing the physiological parameters between the ND-PBS, HFD-PBS, HFD-EcN, and HFD-EcN-2776 groups. a** The serum concentration of IFN-γ, IL-6, and LPS in the four mice groups at day 28 and 70. **b** The concentration of blood urea nitrogen in the blood of four mice groups. Data is presented in mean ± SEM. The statistical significance between different groups is analyzed by one-way ANOVA (**P* < 0.05, ***P* < 0.01, ****P* < 0.001).





**Figure S5.** **The gut microbial alterations of HFD-fed mice after oral administration of EcN-2776. a** The average Good’s coverage of each sample in the ND-PBS, HFD-PBS, HFD-EcN, and HFD-EcN-2776 groups. **b** LEfSe analysis to characterize the taxonomic differences of gut microbiota between the four mice groups. LDA score cut-off was set as 2.0 (P < 0.05). **c** Relative abundance of ASVs assigned at phylum level. **d** The relative abundance of *Akkermansiaceae*, *Bacillaceae*, *Lactobacillaceae*, unclassified_c_*Bacilli*, *Streptococcaceae*, and *Peptostreptococcaceae*. **e** The relative abundance of *Akkermansia* and *Alistipes*. Data is presented in mean ± SEM. The statistical significance between different groups is analyzed by one-way ANOVA (**P* < 0.05, ***P* < 0.01, ****P* < 0.001).

**
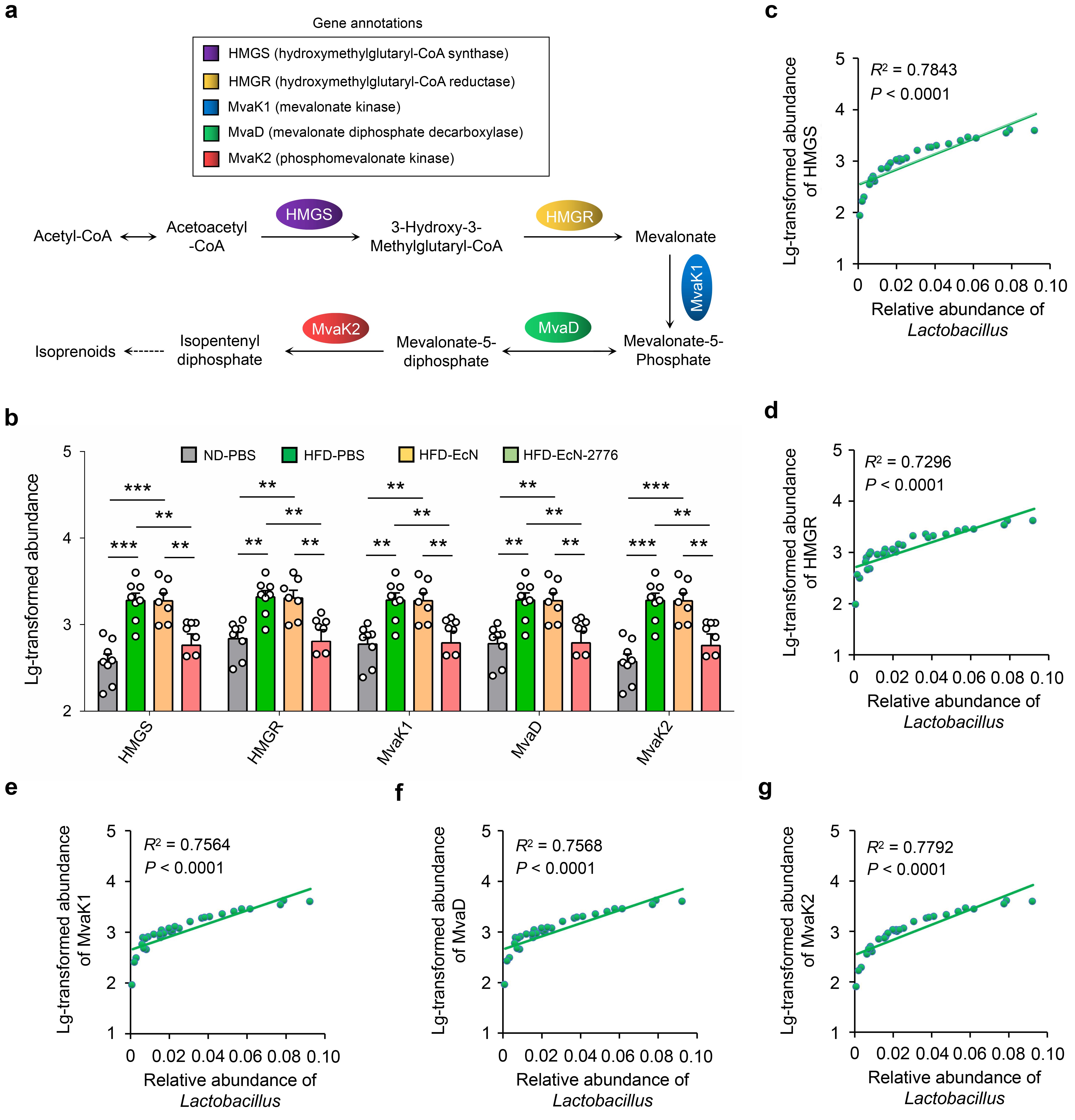
**

**Figure S6. The Lg-transformed abundance of enzymes in the mevalonate (MVA) pathway and their correlations with *Lactobacillus.* a** Schematic diagram showing the five enzymes (HMGS, HMGR, MvaK1, MvaD, and MvaK2) in the mevalonate pathway of *L. murinus*. **b** The Lg-transformed abundance of five enzymes (HMGS, HMGR, MvaK1, MvaD, and MvaK2) in the mevalonate pathway of *L. murinus*. Linear regression analysis is used to assay the correlations between the Lg-transformed abundance of HMGS (**c**), HMGR (**d**), MvaK1 (**e**), MvaD (**f**), and MvaK2 (**g**) and the relative abundance of *Lactobacillus* in the four mice groups. Data is presented in mean ± SEM. The statistical significance between different groups is analyzed by one-way ANOVA (***P* < 0.01, ****P* < 0.001).


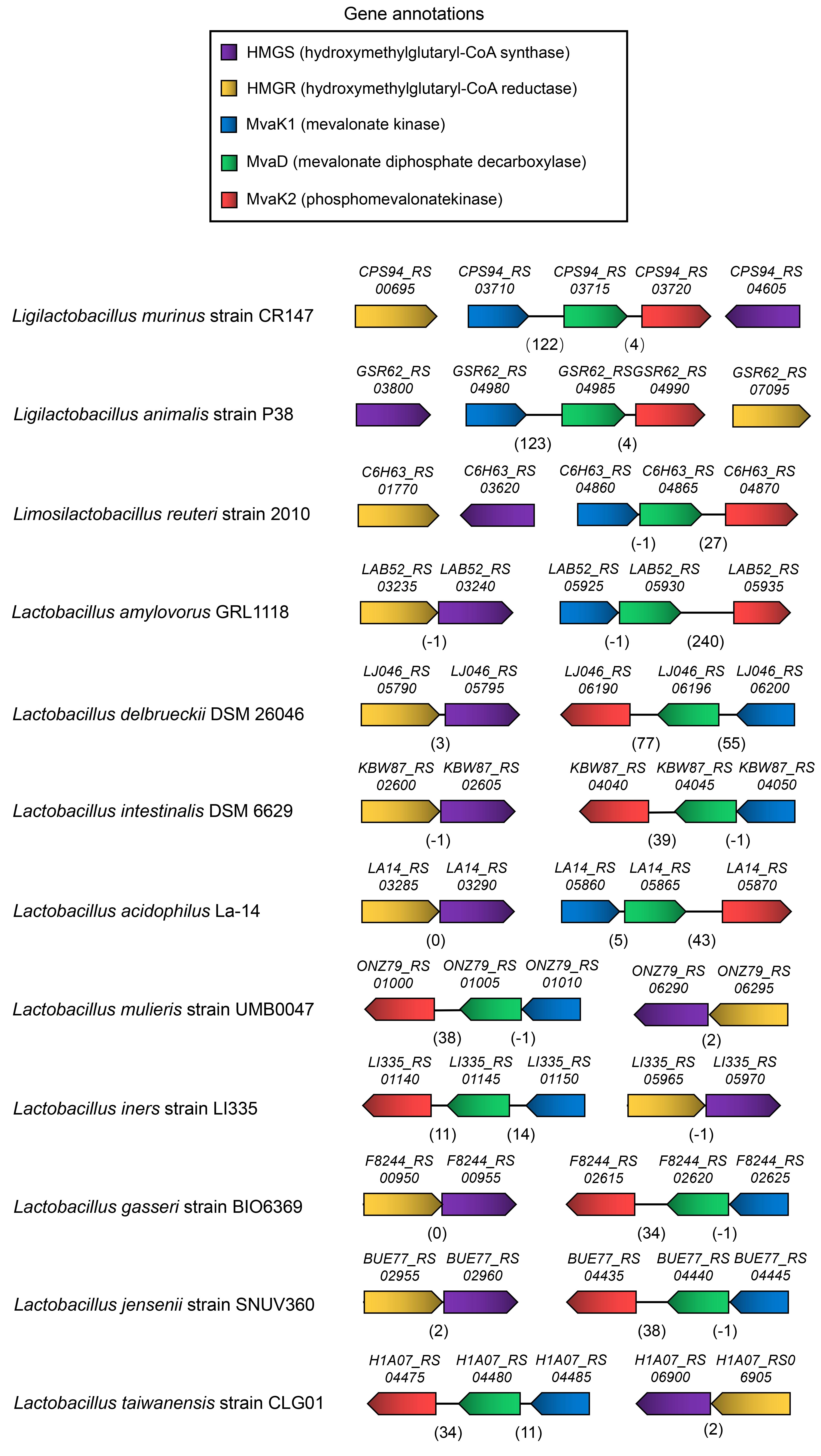


**Figure S7. Identification of the homologous protein of five mevalonate pathway-associated enzymes in various *Lactobacillus* microbes.** Five mevalonate pathway-associated enzymes were labeled as different colors.


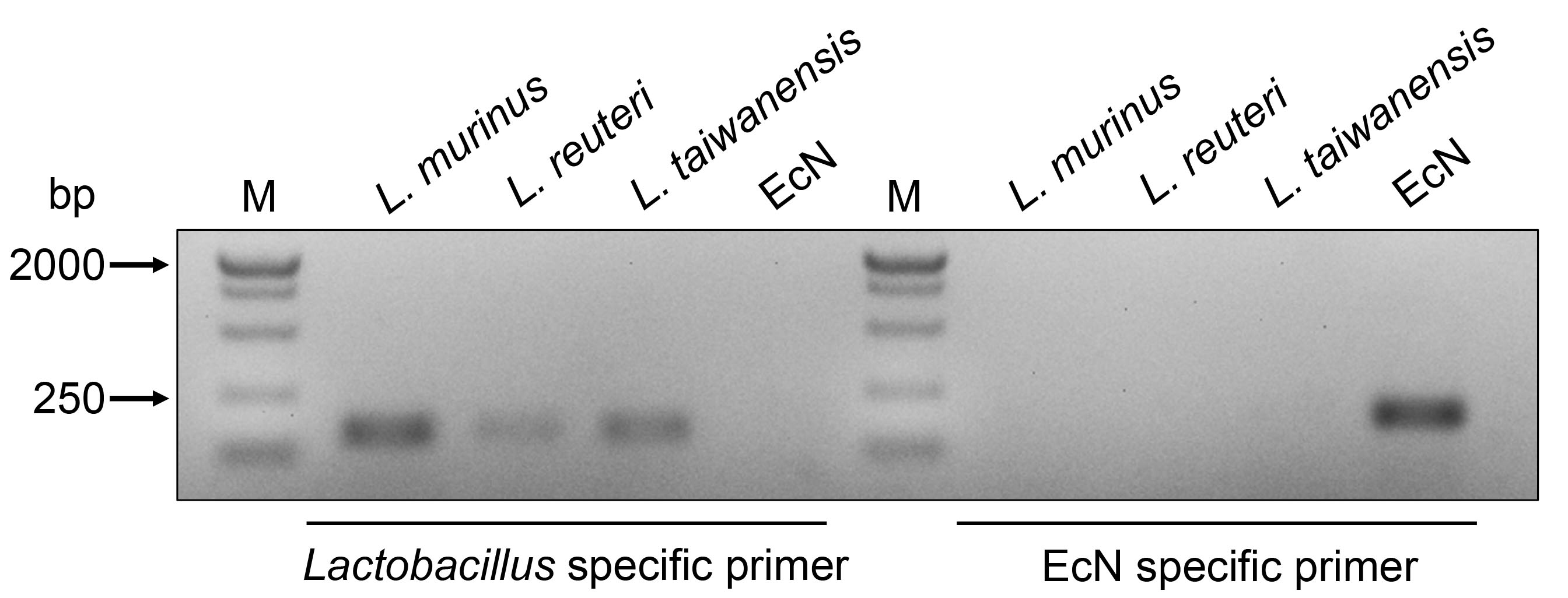


**Figure S8. PCR-based verification of specific primers for three *Lactobacillus* microbes and EcN.**


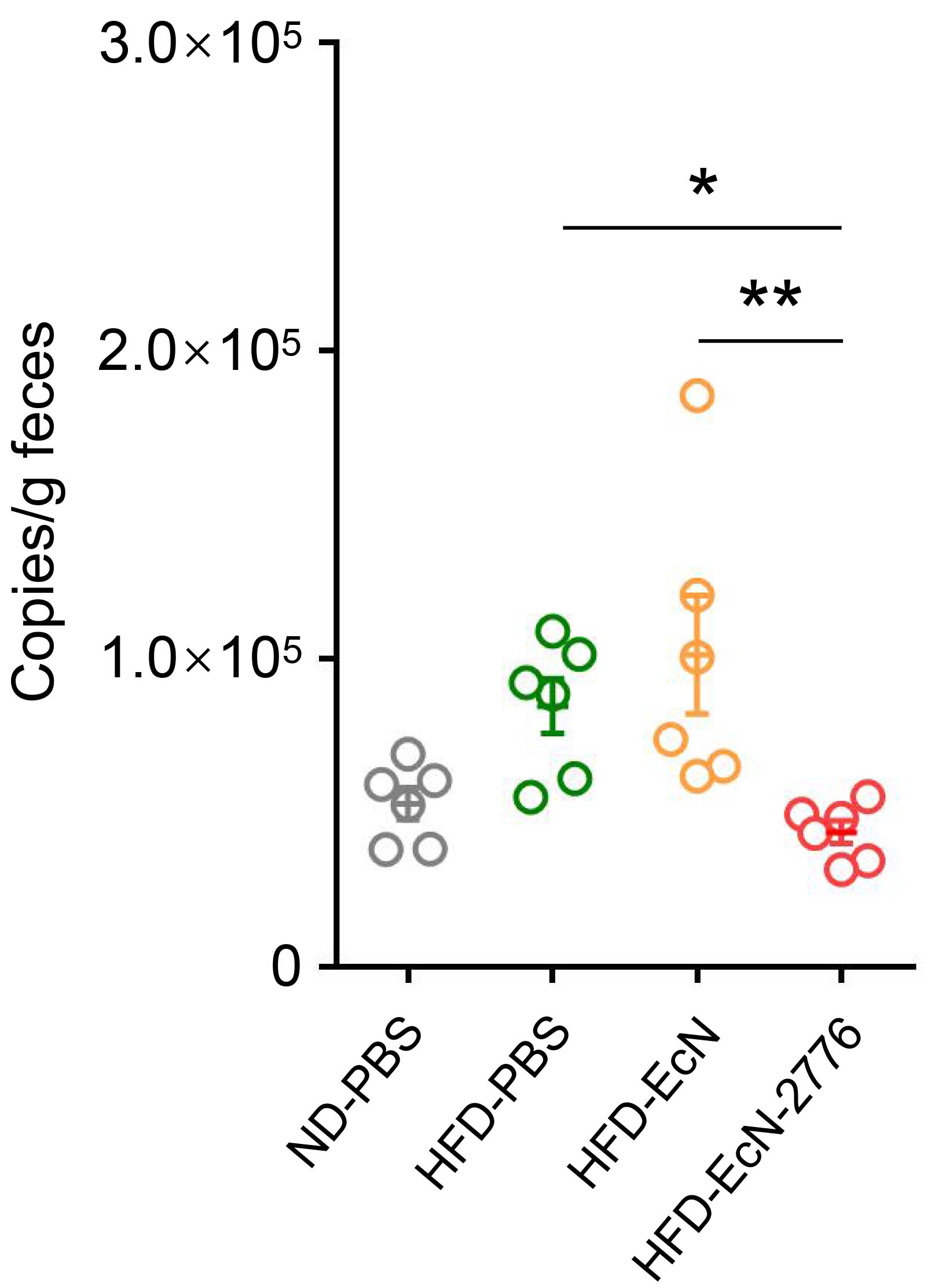


**Figure S9. The absolute abundance (copies/g feces) of *L. murinus* in the gut microbiome of ND-PBS, HFD-PBS, HFD-EcN, and HFD-EcN-2776 groups.** Data is presented in mean ± SEM. The statistical significance between different groups is analyzed by one-way ANOVA (**P* < 0.05, ***P* < 0.01).


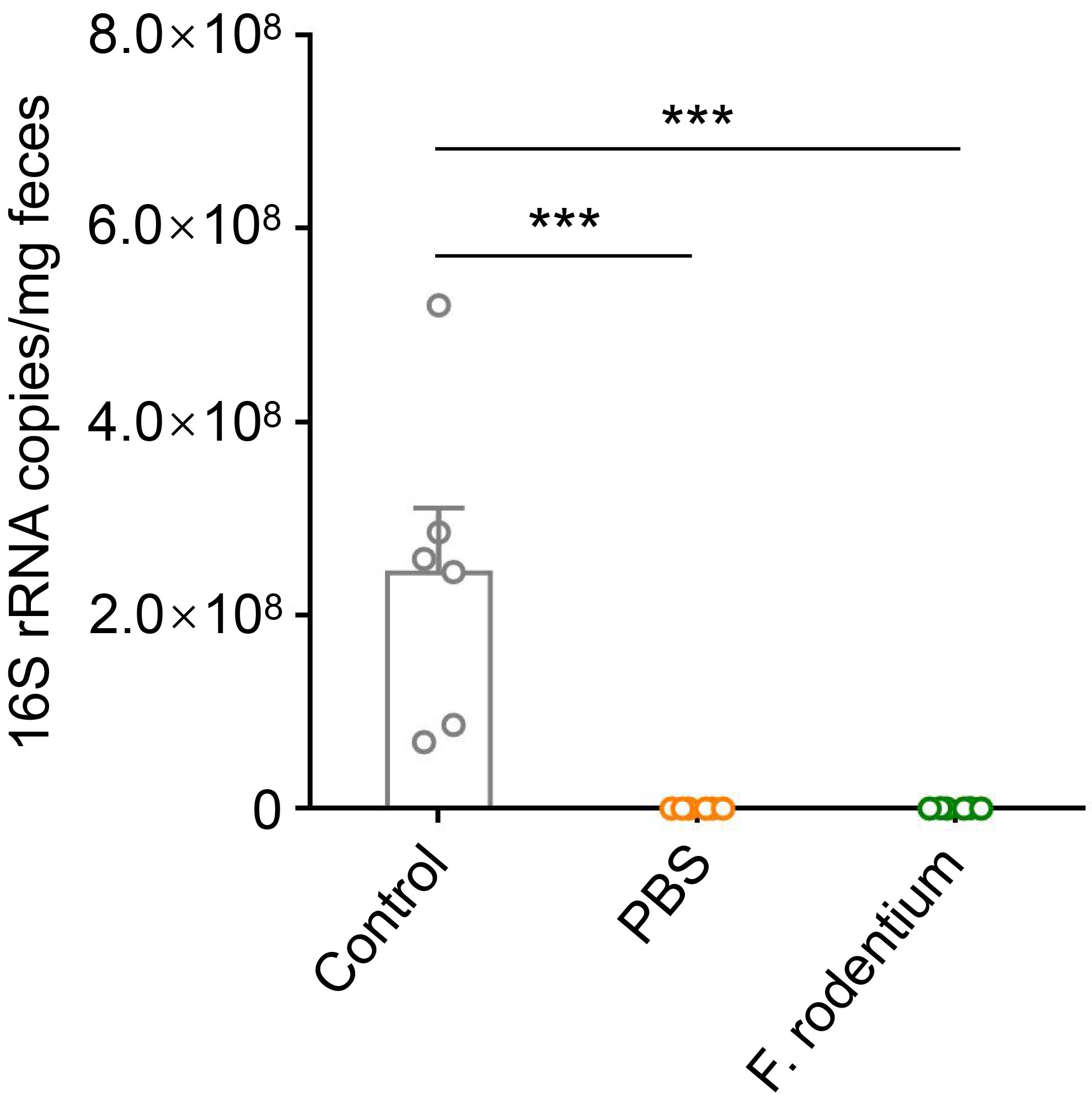


**Figure S10. Verification for the clearance of gut microbes in the ABX-treated mice groups.** The total bacterial loads of gut microbiome in the control, PBS and *F. rodentium* groups were assayed by 16S rRNA quantitative PCR. The control group represented the high-fat diet-fed mice without antibiotic treatment. Data is presented in mean ± SEM. The statistical significance between different groups is analyzed by one-way ANOVA (****P* < 0.001).


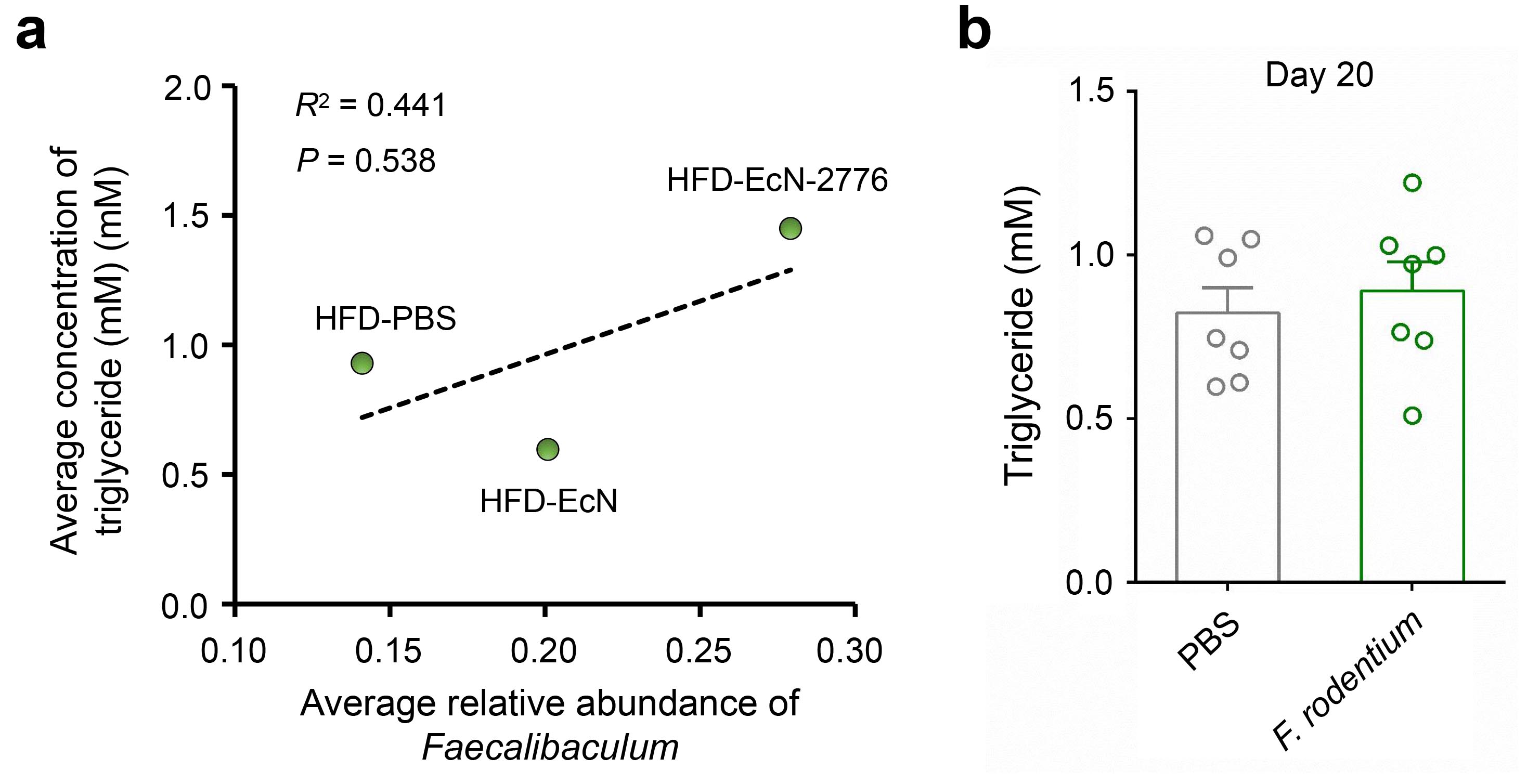


**Figure S11. The impact of *F. rodentium* administration on the lipid metabolism of HFD-fed mice. a** Linear regression analysis for the correlation between the average concentration of triglyceride and the average relative abundance of *Faecalibaculum* in the PBS, EcN, and EcN-2776 groups. **b** The concentration of serum triglyceride between the PBS and *F. rodentium* groups at day 20.

**Table S1.** **Oligonucleotides used in this study.**

| Oligonucleotides name | Sequence (5′-3′) | Description |
| --- | --- | --- |
| Plasmids construction |  |  |
| FliC 5′-s | CACGTTTCCTGAGGAACCGGTACCGTCGACGCGATTTCCTTTTATCTTTC | Forward primer for “FliC 5′ UTR-FliC 20 amino acids” fragment |
| FliC 20-a | GTGGTGGTGGTGGTGGTGCCCGGGCTTGTTGATATTATTTTGAG | Reverse primer for “FliC 5′ UTR-FliC 20 amino acids” fragment |
| His6-s | CACCACCACCACCACCACGACGACGACGACAAAATGAAAAAGAAAGTGTGTGTTC | Forward primer for “His6-enterokinase cleavage site-Lmo2776” fragment |
| Lmo2776-a | GTTAATCAGGTTACGGCGAATCGATTTATTTGGTGGCCCAGTTCGC | Reverse primer for “His6-enterokinase cleavage site-Lmo2776” fragment |
| FliC 3′-s | TCGCCGTAACCTGATTAACTGAG | Forward primer for “FliC 3′ UTR” fragment |
| FliC 3′-a | GGCAGGATGTTTCGTAACCATGGGATAAACAGCCCTGCGTTATATG | Reverse primer for “FliC 3′ UTR” fragment |
| Quantitative PCR assay |  |  |
| *P. copri*-s | TCGCTGACATGAGCGATAAC | Forward *P. copri*-specific primer |
| *P. copri*-a | CCGTTGGCACTACCTTCATT | Reverse *P. copri*-specific primer |
| EcN-s | CCGCGTGTATGAAGAAGGCCTTC | Forward EcN-specific primer |
| EcN-a | GACTTAACAAACCGCCTGCGTG | Reverse EcN-specific primer |
| Lacto-s | TTGATTGCTGATGCAATGGAAAA | Forward *Lactobacillus*-specific primer |
| Lacto-a | TCGTTATCAGTTACCATGTATTG | Reverse *Lactobacillus*-specific primer |
| EcN-s1 | AGTTGCCTCTAAAGCGAACG | Forward EcN-specific primer |
| EcN-a1 | ACGGTACGGACAGCGCTTTC | Reverse EcN-specific primer |
